# Localized immunomodulation with cytokine-producing cells to mitigate foreign body responses in rodents and a nonhuman primate

**DOI:** 10.1126/sciadv.aec7053

**Published:** 2026-08-05

**Authors:** Boram Kim, Dilrasbonu Vohidova, Amanda Nash, Yuen San Chan, Samantha Fleury, Shravani Deo, Danna Murungi, Peter D. Rios, Ira Joshi, Hafsa Nasir, Daisy Lopez, Mor Sela Golan, Cassidy Hart, Jose Oberholzer, H. Courtney Hodges, Omid Veiseh

**Affiliations:** ^1^ Department of Bioengineering, Rice University, Houston, TX, USA.; ^2^ Department of Molecular and Cellular Biology and Center for Precision Environmental Health, Baylor College of Medicine, Houston, TX, USA.; ^3^ CellTrans Inc., Chicago, IL, USA.; ^4^ Department of Visceral and Transplant Surgery, University of Zurich, University Hospital Zurich, Zurich, Switzerland.; ^5^ Rice Biotechnology Launch Pad, Rice University, Houston, TX, USA.

## Abstract

The efficacy of cell-based therapeutics is often compromised by host immune recognition of implanted cells and biomaterials, resulting in fibrotic encapsulation and loss of function. Here, we address this challenge with an immunomodulatory cell-based therapy, in which alginate-encapsulated retinal pigment epithelial cells continuously secrete cytokines to locally modulate the implant microenvironment. In a healthy rodent model, the localized production of interleukin-10 (IL-10) or IL-12 from encapsulated cytokine-producing cells prevented foreign body response to alginate capsules. Mechanistically, treatment was associated with reduced expression of profibrotic genes and immune shifts consistent with macrophage and T cell regulation, supporting a cytokine-mediated mitigation of foreign body response. In a diabetic murine model (streptozotocin-induced C57BL/6J), coimplantation of human islets with IL-10–producing cells attenuated pericapsular fibrosis, preserved islet viability, and restored normoglycemia for up to 100 days (4.76 times longer than islets alone). Notably, IL-10–producing cells were also effective in enabling the durability and function of encapsulated cells in a healthy nonhuman primate, showing translational feasibility. Collectively, these findings suggest that localized cytokine delivery can reduce fibrotic encapsulation and support durable graft function, offering a path to lessen reliance on systemic immunosuppression in islets transplantation and other implantable biomaterial therapies.

## Teaser

Encapsulated IL-10–producing cells locally suppress fibrosis and extend graft function in rodent models and a nonhuman primate.

## INTRODUCTION

Biomaterials are at the forefront of advances in regenerative medicine with applications in medical implants, surgical devices, drug delivery systems, cell-based therapeutics, wound healing, and more. However, one of the biggest remaining challenges for biomaterials is overcoming the foreign body response (FBR), a naturally occurring immunological reaction to implanted “nonself” materials that can culminate in fibrotic encapsulation and loss of function (*1*–*4*). Following implantation, innate immune recognition triggers a coordinated cascade—acute inflammation, macrophage recruitment and activation, foreign body giant cell (FBGC) formation, and ultimately fibroblast-driven extracellular matrix deposition—that physically and functionally isolates the implant from surrounding tissue (*2*–*5*). Macrophages are major mediators of this response: If the foreign material is small enough, then macrophages may attempt to eliminate the material via phagocytosis. However, for most implantable devices and biomaterial depots, macrophages instead accumulate and can fuse into multinucleated FBGC in an effort to degrade or eliminate the material (*3*). When the material persists, macrophage-derived signals recruit and activate fibroblasts, driving the deposition of dense collagenous matrix around the implant (*6*–*8*). The pericapsular fibrotic overgrowth is a major mechanism of failure for implanted biomaterials and encapsulated cell therapies. While material properties (e.g., surface chemistry, charge, topography, material composition, and wettability) can influence the magnitude and kinetics of these responses (*1*, *5*, *8*–*11*), there remains a need for strategies that directly reprogram the immune-fibrotic microenvironment at the implant interface without introducing systemic immunosuppression. Beyond innate immunity, adaptive immunity—including T cell polarization—can shape biomaterial outcomes, as distinct helper T cell and regulatory T cell programs influence cytokine cues that regulate macrophage activation states and fibrotic remodeling at the implant interface (*5*, *12*, *13*).

Because macrophages orchestrate these events through cytokine signaling, tuning the local cytokine environment is a rational way to modulate FBR. Within this cytokine network, interleukin-10 (IL-10) limits macrophage activation, promotes regulatory T cells (T_reg_ cells), and has been shown in preclinical implant models to attenuate fibrosis (*14*). By contrast, IL-12, while classically pro-inflammatory through T helper 1/interferon-γ (IFN-γ) pathways, can exert tissue- and context-dependent antifibrotic effects via IFN-γ–mediated antagonism of transforming growth factor–β (TGF-β) signaling (*15*, *16*). To date, most cytokine therapies in preclinical and clinical settings have been delivered systemically (e.g., intravenous/subcutaneous dosing or systemic depots), approaches that are often constrained by short half-life, peak systemic exposure, and the need for repeated dosing (*17*–*19*). These practical limitations motivate compartmentalized delivery strategies that achieve sustained cytokine exposure at the implant site while minimizing systemic immune perturbation and associated safety risks.

**Fig. 1.**
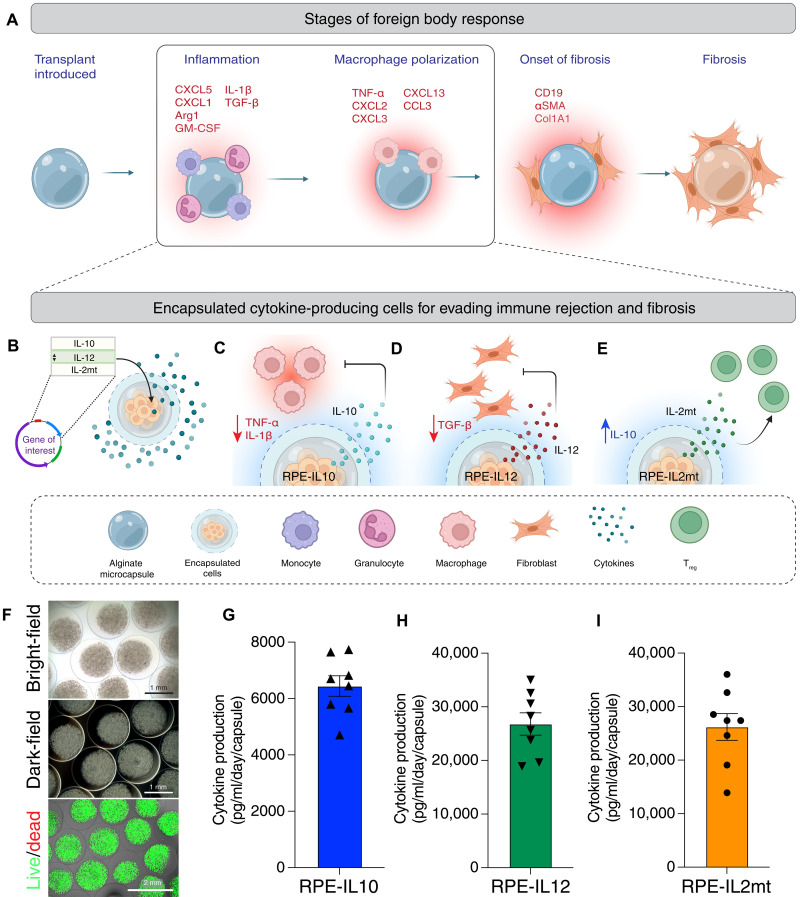
Cytokines released from encapsulated engineered cells modulate the FBR. (**A**) Schematic of FBR progression in alginate microcapsules, illustrating key immune cells, cytokines, and chemokines involved in the FBR based on previously reported data (*3*, *37*). FBR is initiated by protein adsorption, triggering early inflammation, followed by infiltration of granulocytes and monocytes at the implant site. Granulocytes express elevated levels of CXCL5 and IL-1β, while monocyte-derived macrophages secrete granulocyte-macrophage colony-stimulating factor (GM-CSF), arginase-1 (Arg1), TGF-β, and CXCL1, which contribute to fibrotic signaling. As fibrosis progresses, TGF-β expression increases, alongside chemokines such as CXCL2, CXCL3, CXCL13, and CCL3 that promote immune cell recruitment and macrophage polarization. In an attempt to phagocytose the implant, macrophages fuse into FBGCs. High local TGF-β levels drive FBGC formation and the conversion of fibroblasts into myofibroblasts, marked by the increased presence of α–smooth muscle actin (αSMA) and collagen 1A1 (Col1A1)–positive cells over time, indicating the onset of fibrosis. (**B**) Human RPE cells engineered to locally secrete therapeutic cytokines. (**C**) IL-10 suppresses macrophage activation by inhibiting pro-inflammatory cytokine production. (**D**) IL-12 promotes IFN-γ production, which down-regulates TGF-β and restricts myofibroblast differentiation. (**E**) IL-2mt selectively expands IL-10–producing regulatory T cells (T_reg_ cells) due to its reduced affinity for the IL-2Rβγ receptor. (**F**) Bright-field (top), dark-field (middle), and live/dead fluorescence (bottom) images of encapsulated cytokine-producing cells; live cells appear green, and dead cells appear red. (**G** to **I)** Cytokine secretion levels from encapsulated engineered RPE cells over 24 hours measured via ELISA: RPE-IL10 (G**)**, RPE-IL12 (H), and RPE-IL2mt (I). All the schematics were created in BioRender. B. Vohidova (n.d.); https://BioRender.com/ii4lozi.

Localized immunomodulation via the administration of exogenous cytokines has been highlighted recently as a promising method of altering the immunological landscape or response/function within an isolated cavity, such as the abdominal cavity. We use local cell delivery using alginate capsules, a well-studied carrier with mild gelation, biocompatibility, and tunable permeability (*3*, *6*, *8*, *20*, *21*). Despite physical barriers restricting direct cellular contact between host and donor cells, alginate capsules are permeable to secreted factors, which can be recognized as foreign antigens by the recipient’s immune system, triggering an alloreactive immune response (*22*, *23*). This may trigger the FBR, ultimately leading to fibrotic capsule formation and graft failure (Fig. 1A) (*24*–*29*). Building on our prior work, we previously validated an alginate-encapsulated retinal pigment epithelial (RPE) “cytokine factory” platform in which RPE cells secreted IL-2 for local cancer immunotherapy (*21*, *30*). While IL-2 itself is not antifibrotic and that application targeted T cell activation, those studies established the platform’s biocompatibility, manufacturability, containment, and capacity for sustained in vivo cytokine release. Here, we repurpose the same modular delivery system to release antifibrotic cytokines for FBR mitigation in the context of islet transplantation. These localized delivery strategies may be particularly valuable for improving the clinical utility of islet transplantation, especially in light of recent advances such as the Food and Drug Administration (FDA) approval of Lantidra (*31*, *32*) and Vertex’s ongoing Phase 3 trials of stem cell–derived islets (NCT04786262 and NCT06832410), both of which currently rely on systemic immunosuppression. In contrast, local immune modulation enables precise control over the immune response at the graft site, reducing systemic toxicity while enhancing graft protection and long-term function.

In this study, we developed an immunomodulatory cell platform, adapted from our validated IL-2 “cytokine factory” system (*21*, *30*), that uses engineered RPE cells to constitutively secrete immunomodulatory cytokines into the local microenvironment. Encapsulation in alginate protects the cells from direct host interaction while enabling precise cytokine delivery for effective immune modulation. We selected human RPE cells due to their favorable clinical experience, low immunogenicity, and high secretory capacity (*21*, *30*, *33*). Specifically, we engineered RPEs to secrete IL-10, IL-12, or an IL-2 mutein (IL-2mt) (Fig. 1B). IL-10 limits macrophage activation and dampens pro-inflammatory mediators such as tumor necrosis factor–α and IL-1β (Fig. 1C) (*14*); IL-12 can counter-regulate profibrotic TGF-β programs via IFN-γ (Fig. 1D) (*15*, *16*); and IL-2mt, an engineered IL-2 variant with altered receptor-binding, preferentially expands T_reg_ cell through reduced IL-2Rβγ engagement, which can inhibit fibroblast proliferation and potentially prevent or delay fibrotic capsule formation (Fig. 1E) (*34*–*36*). We evaluated this platform for pericapsular fibrosis control around encapsulated islets in streptozotocin (STZ)–induced C57BL/6J mice and assessed translational feasibility in a pilot nonhuman primate (NHP) study with safety and pharmacokinetic monitoring of localized IL-10 delivery. Together, these studies test whether localized cytokine production can mitigate fibrotic encapsulation and support durable graft function while reducing reliance on systemic immunosuppression. This localized immunomodulatory approach presents a clinically translatable strategy not only to improve the durability and safety of islet transplantation but also to broadly address immune-mediated fibrosis and rejection in implanted biomaterials and potentially to treat other autoimmune diseases through targeted, site-specific immune regulation.

## RESULTS

### Fabrication of cytokine-producing cells

To evaluate the efficacy of cytokine-producing RPE cells in preventing biomaterial-induced fibrosis, three distinct cytokines with immune-modulating potential (e.g., IL-10, IL-12, and IL-2mt) were selected. RPE cells underwent genetic modification to express a specific immunomodulatory cytokine through the PiggyBac transposon system. This system enables swift prototype development by substituting the gene of interest while preserving the optimized backbone. We refer to encapsulated cytokine-producing RPE cells as RPE-IL10, RPE-IL12, or RPE-IL2mt, depending on the specific cytokine they were engineered to produce. The engineered cells were encapsulated, as previously described (*21*, *30*), in 1.5-mm alginate capsules containing ∼10,000 cells per capsule, showing highly viable cells after encapsulation (Fig. 1F). Following encapsulation, cytokine secretion from preimplant capsules was confirmed using enzyme-linked immunosorbent assay (ELISA) after 24 hours of incubation (Fig. 1, G to I). RPE-IL10 exhibited a production rate of 6,443 pg/ml per day per capsule, RPE-IL12 yielded 26,794 pg/ml per day per capsule, and RPE-IL2mt produced 26,197 pg/ml per day per capsule.

### Local delivery of anti- or pro-inflammatory cytokines can mitigate fibrotic overgrowth on biomaterial surfaces

**Fig. 2.**
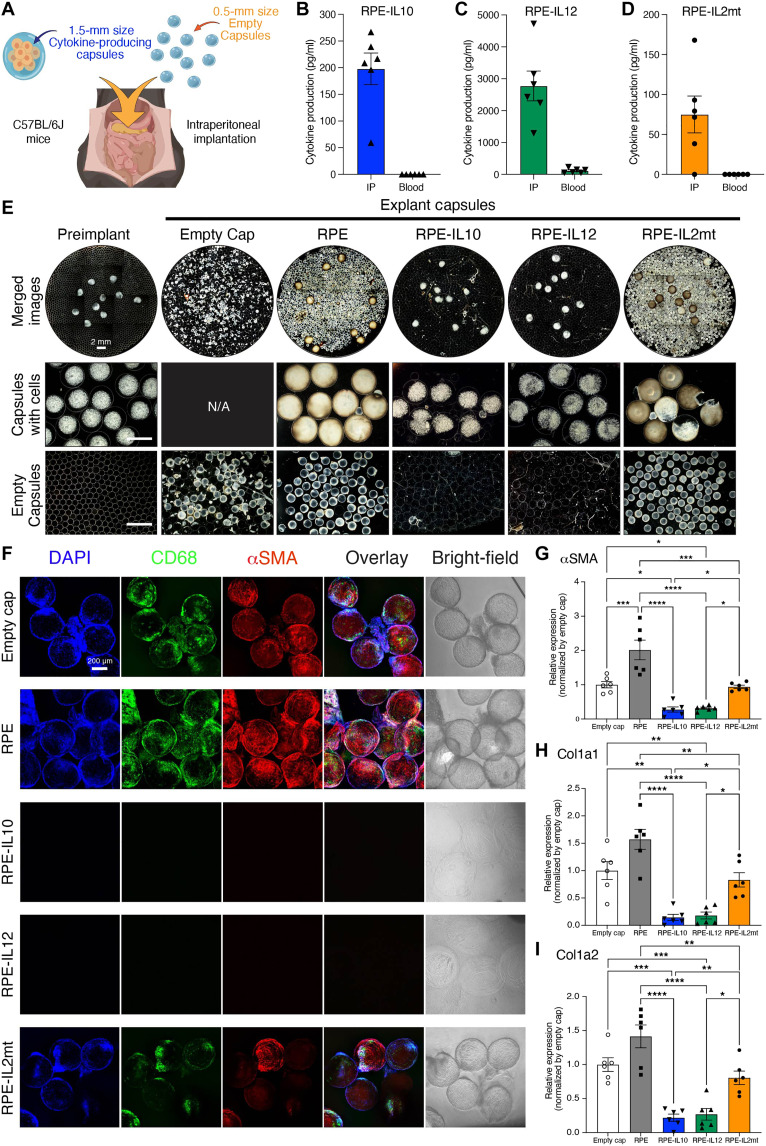
Codelivery of cytokine-producing capsules can mitigate fibrotic tissue overgrowth on biomaterials in short term. (**A**) Schematic of local cytokine delivery in intraperitoneal space of C57BL/6J mouse for 1 month. (**B** to **D**) Cytokine productions in local (intraperitoneal) and systemic (blood) levels after 1 month implantations of RPE-IL10 (B), RPE-IL12 (C), and RPE-IL2mt (D) groups, respectively (*n* = 6). (**E**) Representative dark-field images of pre-implant and explanted capsules. Scare bar, 2 mm. N/A, not applicable. (**F**) Immunofluorescence images of explanted microcapsules. 4′,6-diamidino-2-phenylindole (DAPI), blue; CD68, green; αSMA, red. Scale bar, 200 μm. (**G** to **I**) RT-qPCR analysis to compare fibrotic gene expressions. αSMA (G), Col1a1 (H), and Col1a2 (I) normalized to empty capsule control. All graph bars are means ± SEM of biological replicates (*n* = 6). Two-way analysis of variance (ANOVA) with Bonferroni correction was used for statistical analysis (****P < 0.0001, ***P < 0.0002, **P < 0.002, and *P < 0.033).

To evaluate the antifibrotic effects of cytokine-producing capsules, a mix of large (1.5 mm) and small (0.5 mm) capsules were implanted in the intraperitoneal space of healthy C57BL/6J mice. Large capsules contained cytokine-producing cells, delivering a controlled, low-dose treatment (100,000 cells per mouse) in a minimal volume. The 0.5-mm microcapsules, known to trigger immune responses (*3*), were included to create a pro-fibrotic environment while remaining suitable for future islet encapsulation studies. Each mouse received 10 large (1.5 mm) cytokine-producing RPE capsules (10,000 cells per capsule; ∼100 μl in total) coimplanted with ∼400 μl of empty 0.5-mm microcapsules into the intraperitoneal space (∼500-μl total capsule volume per mouse; Fig. 2A). This design ensured cytokine delivery in a minimal implant volume while allowing the assessment of antifibrotic effects against a robust immune response.

First, the concentration of cytokines in the local (IP space) and systemic cavity (blood) was assessed 1 month after implantation to determine the biodistribution of our cytokine-producing capsules (Fig. 2, B to D). Minimal levels of cytokines were detected in the bloodstream for all three cytokine-producing capsules, suggesting that the administered cytokines were contained within the local cavity. RPE-IL10 (Fig. 2B) and RPE-IL12 (Fig. 2C) exhibited high local concentrations in intraperitoneal fluids (∼195 and ∼2625 pg/ml, respectively), indicating sustained cytokine production in the local space post–1-month implantation. RPE-IL2mt (Fig. 2D) did not demonstrate high local concentration (∼75.5 pg/ml). Subsequently, all microcapsules were retrieved and analyzed with microscopy imaging for fibrotic capsule deposition on their surfaces (Fig. 2E and fig. S1). The groups containing nonengineered RPE or RPE-IL2mt displayed highly packed fibrotic overgrowth on surface of the capsules in all mice, as evidenced by dark-field images. In contrast, the RPE-IL10 and RPE-IL12 groups showed minimal overgrowth on the 1.5-mm cell containing capsules and the empty microcapsules, suggesting that the presence of IL-10 or IL-12 played a role in mitigating the fibrotic process. The empty small-capsule group exhibited fibrotic overgrowth despite the absence of encapsulated cells, consistent with prior reports that alginate microcapsules of this size can trigger robust FBRs (*3*). Explanted RPE-containing capsules were stained with a live/dead assay (live, green; dead, red) to assess viability (fig. S1). In the RPE and RPE-IL2mt groups, green fluorescence was predominantly observed on the capsule surface, likely reflecting viable cells within the fibrotic overgrowth tissue rather than encapsulated RPE cells. In contrast, the IL-10 and IL-12 groups showed minimal surface fibrosis, allowing viable encapsulated RPE cells to be more readily visualized within the capsules. In addition, a high-dose condition was evaluated by loading engineered RPE cells at 40,000 cells per capsule (total 400,000 cells per mouse). RPE, RPE-IL10, and RPE-IL2mt groups were compared 1 month postimplantation, with 0.5-mm microcapsules codelivered as in the low-dose condition (10,000 cells per capsule). High-dose IL-12 was not included due to dose-limiting cytotoxicity (*33*). Under high-dose conditions, RPE-IL10 again exhibited effective fibrosis prevention, showing minimal overgrowth on explanted capsule surfaces relative to RPE controls (fig. S2). RPE-IL2mt did not achieve the level of fibrosis prevention observed with IL-10 despite higher cytokine production. On this basis, subsequent studies were conducted under the low-dose regimen to minimize implanted cell burden while retaining antifibrotic efficacy.

Next, immunofluorescent imaging was used to evaluate the extent of fibrotic overgrowth and cellular deposition on the surface of the explanted capsules (Fig. 2F). Retrieved microcapsules were stained with α–smooth muscle actin (αSMA), a myofibroblast marker, and CD68, a macrophage marker. Results were consistent with dark-field imaging, revealing highly visible fibrotic marker expression on the capsule surface of the empty cap, RPE, and RPE-IL2mt groups but not RPE-IL10 and RPE-IL12 groups. Last, reverse transcription quantitative polymerase chain reaction (RT-qPCR) was conducted to confirm the expression of fibrosis-related genes using cells collected from retrieved microcapsules (Fig. 2, G to I). The relative expression of αSMA (Fig. 2G), Col1a1 (Fig. 2H), and Col1a2 (Fig. 2I) was compared among the groups, and the results aligned with the microscopic findings. Masson’s trichrome further verified these findings as there was no collagen deposition seen on the microcapsules from RPE-IL10 and RPE-IL12 groups (fig. S3). In contrast, the microcapsules from other groups demonstrated substantial collagen presence demonstrated by dark blue color around the capsules. These results suggest that codelivery of either IL-10 or IL-12–producing capsules significantly reduced fibrosis on empty microcapsules compared to the nonengineered cell delivery group.

**Fig. 3.**
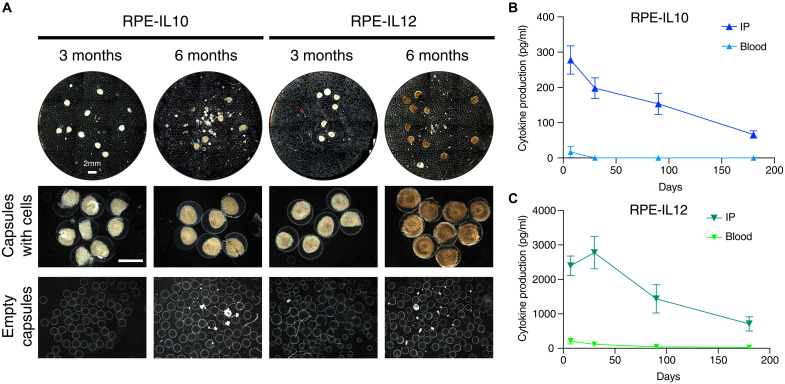
Cytokine-producing capsules prevent long-term fibrotic overgrowth and sustain localized cytokine delivery. (**A**) Representative images of explanted cytokine-producing RPE capsules from the IL-10 and IL-12 groups retrieved at 3 months and 6 months postimplantation, showing minimal fibrotic overgrowth. Scale bar, 2 mm. (**B** and **C**) ELISA quantification of IL-10 (B) and IL-12 (C) concentrations in intraperitoneal (IP) fluid versus blood at 7 days, 1 month, 3 months, and 6 months postimplantation.

To understand the medium and long-term antifibrotic effect of RPE-IL10 and RPE-IL12, we conducted a similar in vivo experiment to the experiment described above. Briefly, 1.5-mm RPE-IL10 or RPE-IL12 capsules were coadministered with empty microcapsules within the intraperitoneal space of healthy C57BL/6J mice for 3 or 6 months (Fig. 3A). At each time point, capsules were retrieved and evaluated under the microscope as described above. All capsules explanted from the RPE-IL10 group demonstrated minimal fibrotic overgrowth on their surface, suggesting sustained prevention of fibrosis (Fig. 3A). Partial fibrotic deposition on the surface of IL-10 capsules was observed in one of six mice at 3 months (fig. S4A) and two of six mice at 6 months (fig. S4B). Similar outcomes were observed in the RPE-IL12 group (Fig. 3A and fig. S5), indicating robust antifibrotic immune modulation (with the exception one mouse of the six from the 6-month post-implantation group).

To assess biocompatibility and downstream effects of cytokine-producing capsule administration, the local and systemic levels of IL-10 and IL-12 were monitored over time via ELISA. Local cytokine secretion was detected at both day 7 (fig. S6) and 1 month after implantation (Fig. 3, B and C). The encapsulated cytokine-producing cells continued to produce detectable local levels of cytokines for up to 6 months when the study was concluded. Systemic levels of both cytokines were undetectable at 3- and 6-month time points, suggesting minimal cytokine leakage into systemic circulation. These comprehensive findings highlight the potential of locally produced cytokines to modulate the local immune response and effectively prevent initiation of fibrosis on biomaterial surfaces.

### IL-10 prevents fibrosis through the suppression of inflammatory response, while IL-12 prevents fibrosis by inhibiting the TGF-β pathway in intraperitoneal immune cells

**Fig. 4.**
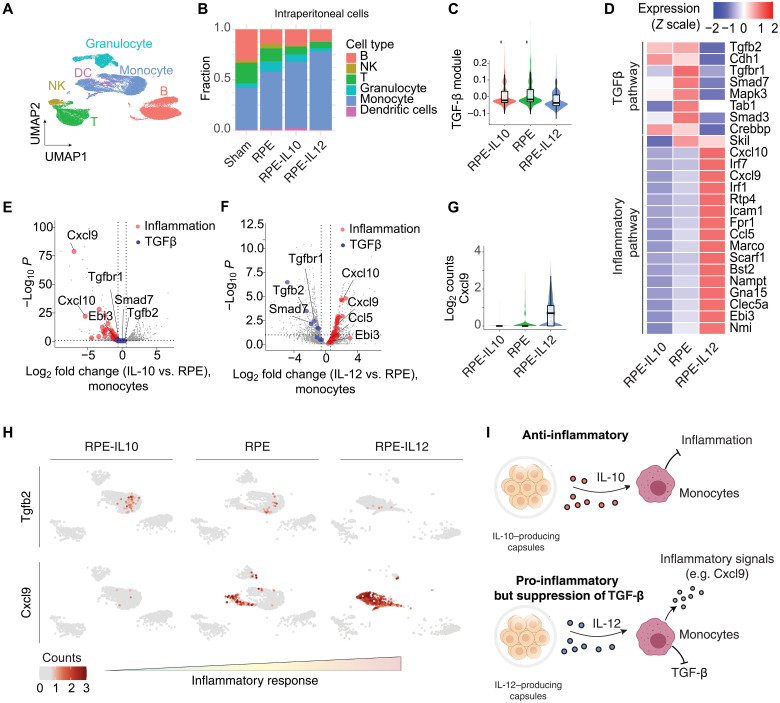
IL-10 prevents fibrosis by suppressing inflammation, while IL-12 prevents fibrosis by inhibiting the TGF-β pathway. (**A**) UMAP embedding of individual cells pooled from all samples. Resulting clusters are classified by immune cell type based on cell-specific expression profiles. (**B**) Composition of local immune cell identities recruited to each implant site. (**C**) Violin plots showing the TGF-β module score in monocytes with RPE-IL10, RPE, and RPE-IL12. TGF-β module score was calculated based on expression levels of RPE-IL12-down-regulated genes in the TGF-β pathway (*P* = 2.9 × 10^−5^ for RPE-IL10 versus RPE, *P* = 2.9 × 10^−16^ for RPE-IL12 versus RPE). (**D**) Heatmap showing *z*-scaled expression levels of representative genes from the inflammatory and TGF-β pathways. (**E** and **F**) Volcano plots showing the differential expression in monocytes treated with RPE-IL10 (E) and RPE-IL12 (F) compared to RPE alone. (**G**) Violin plot showing the inflammatory cytokine Cxcl9 in monocyte with RPE-IL10, RPE, and RPE-IL12 (*P* = 2.9 × 10^−16^ for both RPE-IL10 versus RPE and RPE-IL12 vesus RPE). (**H**) Expression levels of Tgfb2 and Cxcl9 in intraperitoneal immune cells with RPE-IL10, RPE, and RPE-IL12. (**I**) Schematics representing how RPE-IL10 and RPE-IL12 mediated fibrosis suppression. All *P* values are based on cell count. DC, dendritic cell; NK, natural killer.

Next, the underlying mechanism of cytokine-mediated fibrosis prevention was evaluated utilizing single-cell RNA sequencing (scRNA-seq). Briefly, 10 capsules (RPE, RPE-IL10, or RPE-IL12) were coadministered with empty microcapsules within the intraperitoneal space of healthy C57BL/6J mice. Each RPE, RPE-IL10, and RPE-IL12 capsule contained 10,000 cells in a 1.5-mm capsule, while empty microcapsules were fabricated at 0.5-mm size. Sham group mice received 1 ml of saline. A day 7 time point was selected to capture early immune cell recruitment and activation programs that precede fibrotic encapsulation. The capsules were retrieved at day 7 postimplantation, and the cells were collected from the capsule surface and surrounding intraperitoneal fluid or the spleen. This approach allowed evaluation of both local and systemic effects induced by RPE-IL10 or RPE-IL12 in comparison to RPE control capsules upon introduction of foreign materials (in this case, empty microcapsules). Immune cell compositions and expression signatures in each treatment group were analyzed and six distinct clusters using Uniform Manifold Approximation and Projection (UMAP) embedding were identified. Based on standard cell type–specific markers, these clusters corresponded to granulocytes, monocytes (including macrophages), dendritic cells, B cells, T cells, and natural killer (NK) cells (Fig. 4A and fig. S7A). scRNA-seq analysis revealed that the composition of immune infiltrate in local space was significantly altered by cytokine-producing capsules (Fig. 4B). Compared to control RPEs, RPE-IL10 increased the intraperitoneal monocytes by 8.95% (*P* = 1.50 × 10^−12^), while RPE-IL12 increased the intraperitoneal monocytes by 20.89% (*P* = 1.48 × 10^−64^) (table S1). Notably, both cytokines significantly reduced the proportion of granulocytes, T cells, and NK cells present in the intraperitoneal space by at least 3% (*P* < 1.1 × 10^−10^ for all) and mildly altered (increased or decreased) the B cells and dendritic cell proportion by less than 3% (*P* < 0.05 for all) (table S1).

The previous report (*37*) demonstrated that macrophages are indispensable to the fibrotic cascade in response to the implanted biomaterial. Despite that, our results showed that both antifibrotic RPE-IL10 and RPE-IL12 enriched the macrophage-containing monocyte population. To investigate the effect of RPE-IL10 and RPE-IL12 on fibrotic pathways, including inflammatory response and TGF-β (*38*, *39*) pathway in monocytes, differential expression profiles of monocytes treated with each cytokine were compared to RPE alone. Here, the TGF-β pathway was investigated as it was shown to play a vital role in promoting fibrosis (*39*). Examining the expression of the TGF-β module indicated a significant decrease in TGF-β expression in the RPE-IL12 group compared to the RPE control [normalized enrichment score (NES) = −2.1, *P* = 1.0 × 10^−3^) (Fig. 4, C and D, and table S2). However, RPE-IL10 had mild but not statistically significant effects on the fibrotic TGF-β pathway (NES = −0.97, *P* = 0.49) (Fig. 4, C and D, and table S3). In analyzing the effects on the inflammatory pathway (Fig. 4D), it showed that RPE-IL10 significantly suppressed genes associated with the inflammatory response. In contrast, RPE-IL12 significantly promoted genes related to inflammatory response. Looking more closely, RPE-IL10 led to the downregulation of inflammatory Cxcl9 expression in monocytes (*P* < 2.2 × 10^−16^) (Fig. 4E). Flow cytometry further confirmed an increased proportion of anti-inflammatory M2-like macrophages (CD11b^+^CD206^+^) and a reduced proportion of pro-inflammatory M1-like macrophages (CD11b^+^CD86^+^) in the RPE-IL10 group (fig. S8). The closer examination of RPE-IL12 revealed that it down-regulated profibrotic Tgfb2 (*P* = 4.9 × 10^−8^; Fig. 4F) (*40*). As expected, in contrast to RPE-IL10, RPE-IL12 showed a higher expression of Cxcl9 (*P* = 2.9 × 10^−16^) (Fig. 4G). In addition, gene sets related to allograft rejection and antigen processing cross-presentation revealed that RPE-IL10 suppressed genes related to allograft rejection (NES = −1.74, *P* = 2.8 × 10^−4^) and antigen processing cross-presentation (NES = −1.74, *P* = 2.1 × 10^−3^) (table S3). On the other hand, RPE-IL12 upregulated genes related to allograft rejection (NES = 1.32, *P* = 0.004) and antigen processing cross-presentation (NES = 1.72, *P* = 4.0 × 10^−6^) (table S2). The expression of Tgfb2 was decreased in RPE-12 (top graphs), and the expression of Cxcl9 was reduced in RPE-IL10 group (bottom graphs) (Fig. 4H). These results demonstrate that RPE-IL10 and RPE-IL12 exert distinct antifibrotic mechanisms; RPE-IL10 suppresses inflammation, while RPE-IL12 inhibits the TGF-β pathway (Fig. 4I).

Last, the differences in local versus systemic effects of cytokines on the gene expression profiles in the intraperitoneal and spleen in RPE-IL10 or RPE-IL12 groups were compared to RPE group (fig. S7). RPE-IL10 does not significantly affect the gene expression in the spleen related to inflammatory gene sets, including allograft rejection (NES = −1.21, *P* = 0.13) and antigen processing cross-presentation (NES = −1.01, *P* = 0.43), indicating the effect of RPE-IL10 is primarily localized (fig. S7, B and C, and table S4). In contrast, RPE-IL12 significantly promoted inflammatory response, including allograft rejection (NES = 1.64, *P* = 7.8 × 10^−4^) and antigen processing cross-presentation (NES = 2.04, *P* = 6.9 × 10^−6^), and suppress TGF-β pathway (NES = −1.96, *P* = 5.8 × 10^−4^) in splenic monocytes, indicating the effect of RPE-IL12 is systemic (fig. S7, B and C, and table S5). These results concluded that IL-10 is an ideal cytokine to prevent fibrosis with minimal systemic effect on the host’s immune cells.

### IL-10–producing cells prevent fibrosis of encapsulated xenogeneic islets and enable long-term normal glycemic control in an immunocompetent diabetic mouse model

**Fig. 5.**
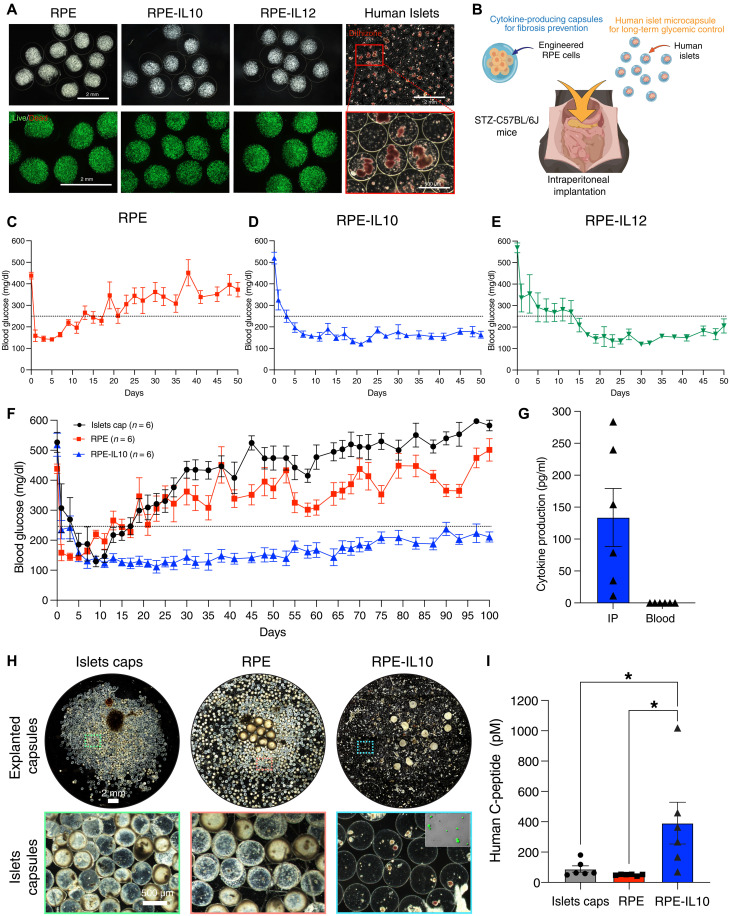
RPE-IL10 capsules codelivered with encapsulated human islets achieved glucose correction in immunocompetent STZ-induced diabetic C57BL/6J mice. (**A**) Representative images of preimplant capsules. Dark-field images of alginate encapsulated 10,000 nonengineered RPE, RPE-IL10, and RPE-IL12 cells per capsule in a 1.5-mm diameter. Top row; scale bars, 2 mm. After encapsulation, RPE, RPE-IL10, and RPE-IL12 demonstrate good viability. Live, green; dead, red. Bottom row; scale bars, 2 mm. Dithizone staining (in red) shows β cells inside the 0.5-mm diameter microcapsules in the last column. Scale bars, 2 mm and 500 µm for top and bottom images, respectively. (**B**) Cytokine-producing RPE capsules are implanted with encapsulated human islets in the intraperitoneal space of STZ-induced diabetic mice. (**C** to **E**) Blood BG levels of RPE (C), RPE-IL10 (D), and RPE-IL12 (E) groups delivered with encapsulated 2000 IEQ islets up to 50 days (*n* = 6). Mice with BG levels below dashed gray lines (250 mg/dl) were considered to be in normoglycemic condition. (**F**) BG levels of RPE and RPE-IL10 capsules with islets microcapsules and islets microcapsules alone for 100 days (*n* = 6). (**G**) IL-10 local concentration (intraperitoneal) and systemic concentration (blood) from the RPE-IL10 group at day 100. (**H**) Representative dark-field images of retrieved islets cap, RPE, and RPE-IL10 groups post–100 days implantation. Scale bars, 2 mm (top) and 500 μm (bottom). Inset in RPE-IL10; live/dead image of explanted islet capsules. (**I**) Human C-peptide levels in serum at day 100 posttransplantation for islets cap, RPE, and RPE-IL10 groups. All error bars denote means ± SEM of biological replicates. One-way ANOVA with Bonferroni correction was used for statistical analysis (*P < 0.033).

To ensure the therapeutic feasibility of our localized delivery strategy, we first confirmed that the presence of RPE-IL10 cells did not undermine the physical or functional integrity of the human donor islets. By coencapsulating these cell lines, we verified that the islets were not adversely affected by their proximity to the cytokine-secreting cells. Moreover, microscopic evaluation confirmed complete cellular containment within the capsules, while static glucose-stimulated insulin secretion (GSIS) assays demonstrated that the islets maintained robust functional potency (fig. S9). To evaluate the efficacy of RPE-IL10 and RPE-IL12 in protecting xenogeneic islets, we coimplanted cytokine-producing capsules in the intraperitoneal space in STZ-induced diabetic C57BL/6J mice together with alginate-encapsulated human islets (2000 islet equivalents; Fig. 5, A and B). RPE, RPE-IL10, or RPE-IL12 cells were encapsulated as described previously, and 10 capsules per mouse were coadministered with 0.4 ml of islet microcapsules. As in the fibrosis study, islet capsules were fabricated at 0.5-mm size to trigger a more robust FBR upon implantation. Following capsule administration, nonfasting blood glucose (BG) levels were markedly decreased and restored to normal glycemia levels in all groups. However, after 3 weeks, the RPE group failed glycemic correction (Fig. 5C), while the RPE-IL10 (Fig. 5D) and RPE-IL12 (Fig. 5E) groups demonstrated continued BG controls in diabetic mice up to 50 days with average BG levels below 250 mg/dl (which is commonly considered as healthy condition in the field).

To assess the durability of glycemic correction associated by cytokine-producing cells, BG levels of the RPE-IL10 group were monitored for 100 days in diabetic mice. RPE group coadministered with human islets (labeled as RPE) and only islets capsule group (labeled as islet cap) were used as controls. RPE-IL12 was not extended due to off-target complications (ascites) observed in a subset of animals, and subsequent analyses therefore focused on IL-10. RPE-IL10 with islet treatment demonstrated robust and extended BG regulation for 100 days compared to islet cap and RPE groups, which both failed before 3 weeks (Fig. 5F). The sustained metabolic benefit in REP-IL10 group coincided with continued local cytokine availability, as IL-10 remained detectable in intraperitoneal fluid at day 100 (Fig. 5G), indicating sustained secretion from the encapsulated cytokine-producing cells. Consistent with a cytokine-mediated attenuation of FBRs, explanted islet microcapsules from RPE-IL10 mice showed minimal pericapsular fibrotic overgrowth with highly viable islets relative to RPE and islets-only controls (Fig. 5H). In addition, the concentration of human C-peptide, a surrogate biomarker for insulin production, was measured from the serum separated from mouse blood at day 100 posttransplantation. Significantly higher levels of C-peptide secretion were detected in the RPE-IL10 group compared with controls, supporting the preservation of islet viability and function (Fig. 5I). Together, these findings support a coherent sequence in which persistent local IL-10 reduces fibrosis, preserves islet function, and sustains durable glycemic control, all achieved without systemic immunosuppression.

### IL-10–producing cells sustain local cytokine production and are well tolerated in an NHP

**Fig. 6.**
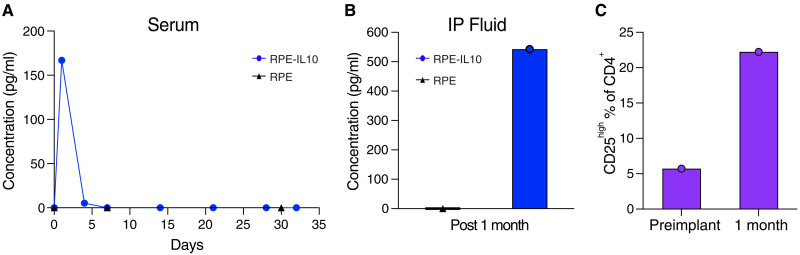
IL-10–producing cells showed sustained release of IL-10 and are well tolerated in a NHP. (**A**) Cytokine levels in the serum were measured over time via ELISA following administration of RPE-IL10 or control RPE capsules in each NHP. (**B**) Cytokine concentrations in the intraperitoneal fluid were measured by ELISA one month after administration of RPE-IL10 or control RPE capsules in each NHP. (**C**) Changes in the frequency of CD4^+^CD25^high^ regulatory T cells in blood were assessed before capsule implantation and after explantation of RPE-IL10 capsules.

To evaluate clinical translatability, cynomolgus macaques received intraperitoneal implantation of alginate-encapsulated cells in a pilot design: One animal received RPE-IL10 capsules (*n* = 1), and one control animal received nonengineered RPE capsules (*n* = 1). This study was not powered for efficacy and was intended to assess feasibility, pharmacokinetics, and tolerability. To assess cytokine leakage into the systemic circulation, cytokine levels in serum were measured. Previously published RPE-IL2 data (*30*) are referenced only for historical context. IL-10 levels showed transient systemic elevation on day 1 postimplantation, becoming undetectable by day 4 (Fig. 6A), while cytokines were undetectable at all time points in the RPE control animal. These findings are consistent with our prior report using RPE-IL2 capsules (*30*), which demonstrated transient IL-2 detection in serum on day 3 but not by day 5, consistent with brief, low-level systemic exposure early after implantation rather than sustained leakage. To assess local cytokine production, intraperitoneal fluid was collected 1 month postimplantation. In contrast to earlier findings with RPE-IL2 capsules (*30*), where cytokine levels were undetectable due to fibrotic encapsulation, IL-10 remained detectable in the RPE-IL10 group, suggesting sustained local cytokine production and reduced fibrosis (Fig. 6B). Pharmacodynamic effects of IL-10–producing cells were evaluated by monitoring CD4^+^CD25^high^ T_reg_ cell frequencies in the peripheral blood. The flow cytometry of peripheral blood mononuclear cells, collected before implantation and after explantation, revealed T_reg_ cell expansion in the blood stream following RPE-IL10 implantation (Fig. 6C). General toxicity indicators—including body weight, body temperature, and platelet count—remained within normal ranges throughout the study (Table 1). IL-10–related toxicities—particularly in the liver, kidneys, and lungs—were also assessed, as elevated IL-10 levels can suppress immune responses and impair organ function (*41*–*44*). The histopathological examination of these organs via hematoxylin and eosin (H&E) staining revealed no abnormalities, which aligns with the reported histology of healthy NHP organs (fig. S10) (*45*). Complete blood counts and blood chemistry showed no significant changes in red blood cell, white blood cell, or lymphocyte counts (table S6). Kidney function was monitored through creatinine, blood urea nitrogen (BUN), and potassium levels (Table 2). Although creatinine levels were slightly below baseline, this was unrelated to treatment, as levels were already low before implantation. BUN and potassium levels remained within healthy ranges. Liver function was assessed by measuring aspartate aminotransferase (AST), alanine aminotransferase (ALT), and gamma-glutamyl transferase (GGT) levels, and no significant changes were observed (Table 3). These findings demonstrate that IL-10–producing cells support sustained, localized cytokine production without systemic exposure or toxicity, highlighting their safety and translational potential.

**Table 1.** General toxicity assessment. Longitudinal monitoring of body weight, body temperature, and platelet count. N/A, not applicable.

	Day 0	Day 1	Day 4	Day 7	Day 14	Day 21	Day 28	Normal range
Body weight (kg)	3.6	3.6	3.5	3.45	3.45	3.8	3.75	N/A
Temperature (°C)	34.3	36.9	37.3	37.5	37.1	37.7	37.6	N/A
Platelet count (×10^3^/μl)	427	349	191	546	529	586	628	154.56–636.2

**Table 2.** Kidney function assessment. Longitudinal analysis of serum creatinine, BUN, and potassium levels.

	Day 0	Day 1	Day 4	Day 7	Day 14	Day 21	Day 28	Normal range
Creatinine (mg/dl)	0.5375	0.5061	0.4969	0.5516	0.5261	0.5459	0.529	0.648–1.62
BUN (mg/dl)	15	12	23	18	20	23	20	5.6–25.2
Potassium (mM)	3.6	3.5	3.8	4	4	3.7	3.9	3.4–7.68

**Table 3.** Liver function assessment. Longitudinal analysis of AST, ALT, and GGT levels.

	Day 0	Day 1	Day 4	Day 7	Day 14	Day 21	Day 28	Normal range
AST (IU/liter)	22	33	17	18	18	35	23	18.4–78
ALT (IU/liter)	20	28	18	18	14	20	23	2.4–33.6
GGT (IU/liter)	131	131	89	73	53	69	94	39.2–134.4

## DISCUSSION

This study presents a substantial advancement in cell-based therapeutics by addressing one of the most pressing challenges: immune rejection and fibrosis of implanted biomaterials and encapsulated cell therapies. By integrating a clinically translatable alginate-based platform with localized immunomodulatory cytokine delivery, our approach supports the robust and prolonged functionality of implanted cell therapeutics. We selected RPE cells as the cytokine-producing source because they are nontumorigenic, contact-inhibited, readily genetically modifiable, and have prior clinical safety experience (*30*, *46*–*49*); consistent with this, our previous report with alginate-encapsulated RPE cells showed no cell division within capsules (*30*). In addition, RPE cell line used here has clinical precedent in an FDA-approved encapsulated cell therapy implant (ENCELTO) for a rare eye disease (*50*), supporting the translational potential of this cell source. Our findings build upon previous efforts to mitigate immune rejection in cell therapy platforms. Earlier strategies focused on chemical surface modifications, implant geometry alterations, or covalent polymer analogs to reduce immune responses and fibrosis (*6*, *8*, *20*, *51*). While these approaches demonstrated promising preclinical results, translating them into clinical practice has proven challenging due to inconsistent outcomes and limited long-term efficacy (*52*, *53*). In parallel, clinical experiences with cytokine delivery by direct injection or slow-release depots have often been constrained by short half-lives, repeated dosing requirements, and systemic exposure-related toxicities (*33*). The delivery of IL-10 and IL-12 through our platform reduced fibrotic deposition in immunocompetent mouse models over 6 months, a substantial improvement compared to prior methods. This effect was achieved without systemic toxicity, as cytokine secretion remained localized at the implantation site. These observations suggest that this cytokine-producing cell platform can reshape cytokine pharmacokinetics toward sustained, local exposure while minimizing systemic leakage, which may be difficult to achieve with bolus injections or conventional depots of cytokines.

The scRNA-seq analysis supports distinct, cytokine-specific mechanisms for fibrotic capsule mitigation. In the intraperitoneal compartment, IL-10 reprogrammed monocytes/ macrophages by down-shifting inflammatory and antigen presentation modules. This local tuning aligns with reduced pericapsular fibrosis and preserved islet function, without implying global immunosuppression. In contrast, IL-12 demonstrated a mechanism of action by modulating the TGF-β pathway, a critical mediator of fibrotic processes. IL-12 delivery led to localized inflammatory responses while simultaneously inhibiting fibrotic capsule formation. This dual action highlights IL-12’s complex immunomodulatory role, as observed in our scRNA-sequencing analysis, which revealed the pleiotropic effects of TGF-β in promoting both anti-inflammatory signaling and fibrosis (*39*, *40*). Despite its localized inflammation, IL-12’s ability to suppress extracellular matrix deposition positions it as a potential target for further investigation.

The inclusion of IL-10 and IL-12 into an alginate-based platform represents a key innovation in local immune modulation. Previous attempts to address graft rejection relied on systemic immunosuppressive therapies or engineered cell types such as cytokine-producing engineered β cells (*36*) or immunomodulatory engineered MSCs (*54*, *55*), which are associated with inconsistencies in cell survival, dose control, and spatial distribution. Other immunomodulatory approaches, such as checkpoint protein-releasing microgels (*56*, *57*), demonstrated short-term efficacy but often required additional immunosuppressive regimens. In comparison, our platform offers several advantages. First, it enables sustained, localized cytokine delivery tailored to specific therapeutic needs. Second, it eliminates the requirement for systemic immunosuppression, thereby minimizing adverse immune-related effects. Third, the platform’s ability to achieve long-term efficacy in preventing xenogeneic graft rejection under stringent immune conditions highlights its translational robustness. We observed concurrent local IL-10 detection, reduced pericapsular fibrosis, and preservation of human C-peptide with durable glycemic control in a diabetic mouse model, supporting a biologically linkage between cytokine delivery, fibrotic tissue mitigation, and function. Collectively, these features distinguish our approach as a transformative advancement in cell delivery platforms.

The clinical implications of this work are particularly promising for T1D and other autoimmune diseases. Treatments aimed at slowing or reversing the fibrotic cascade have targeted modulating myofibroblast activation, reducing inflammation, inhibiting ECM deposition, reducing collagen synthesis, and/or enhancing ECM degradation, and many are now being evaluated in clinical trials. Examples of antifibrotic treatments include drugs targeting (i) the TGF-β pathway, such as pirfenidone, which is an FDA-approved treatment for patients with idiopathic pulmonary fibrosis (*58*); (ii) major growth factor pathways, such as nintedanib, which is a receptor tyrosine kinase inhibitor (*58*); (iii) the Pi3K pathway such as parsaclisib, which is a Pi3Kγ inhibitor (*59*), (iv) the Janus kinase (JAK) pathway such as ruxolitinib, which is JAK1/JAK2 inhibitor that is FDA-approved for patients with myelofibrosis (*60*); and (v) glucose and lipid metabolism such as semaglutide, which is a glucagon-like peptide-1 receptor agonist that is FDA-approved for type 2 diabetes mellitus (*61*). Because many cell types and signaling pathways are implemented in fibrosis, there is no shortage of drug targets to attempt to ameliorate this process. Unfortunately, the side effects of these drugs can be severe and thus remain a major hurdle for drug development. Safer treatments for patients are urgently needed.

IL-10 has been extensively studied for its role in immune modulation, with previous reports demonstrating its ability to suppress insulitis and delay diabetes onset in animal models (*62*, *63*). In humans, elevated IL-10 levels have been associated with improved β cell protection and immune tolerance (*63*). However, prior attempts to deliver IL-10 using viral vectors or recombinant proteins faced challenges, including immunogenicity, toxicity, and rapid cytokine degradation (*64*). Our platform overcomes these limitations by providing long-term, localized IL-10 delivery, ensuring sustained therapeutic effects while minimizing systemic exposure. Notably, therapeutic colocalization of every cytokine-producing capsule with islet capsules is not required in the intraperitoneal cavity; the peritoneum functions as a permissive diffusion compartment in which a relatively small fraction of cytokine-producing capsules can maintain a bioactive cytokine condition that influences the broader graft environment. Mechanistically, IL-10–associated reductions in fibrotic capsule formation are consistent with decreased expression of profibrotic markers and preserved islet function observed at late time points.

Beyond T1D, this local immunomodulatory platform holds potential for other autoimmune, inflammatory, and infectious diseases. For instance, the ability to deliver IL-10 locally could be applied to conditions such as rheumatoid arthritis, inflammatory bowel disease, and organ transplantation, where immune modulation is critical for disease management (*65*–*68*). The platform’s modularity also allows for the incorporation of other cytokines or therapeutic agents, expanding its utility across various medical applications. Future studies exploring these opportunities will further establish the platform as a versatile and clinically translatable solution for fibrosis-related disorders. To assess clinical translatability, we conducted a pilot NHP evaluation focused on feasibility, pharmacokinetics, and tolerability rather than efficacy. In the NHP study, the cytokine-producing cells successfully prevented pericapsular fibrosis and immune rejection of cell therapeutics, mirroring the results observed in mouse models. Notably, the localized delivery of IL-10 maintained cytokine concentrations at the implant site without detectable systemic toxicity post one month implantation, demonstrating its safety in a clinically relevant model.

While this study demonstrates considerable advancements, certain limitations must be addressed in future research. Although IL-10 delivery effectively prevented pericapsular fibrosis and graft rejection in immunocompetent mouse and showed feasibility and tolerability in a pilot NHP study, the NHP findings should be interpreted cautiously given the limited sample size. Also, the systemic effects of IL-12 observed in splenic monocytes raise concerns regarding its long-term safety and immunogenicity. We note that fluid accumulation was not observed with the IL-12 dose in healthy mice, suggesting disease-context interactions in diabetic models; nevertheless, dose titration and temporal control will be required for safe translation. Future work will include refined IL-12 dosing with mechanistic validation, and safety features for IL-10 delivery such as inducible expression or kill-switches to allow on-demand cessation. In addition, while the platform’s success has been demonstrated in mouse models and initially evaluated in NHPs for feasibility, further investigation into disease-specific fibrotic models, such as idiopathic pulmonary fibrosis and liver fibrosis, will help expand its therapeutic applications. Long-term surveillance for infection susceptibility or tumor immunity under sustained IL-10 exposure will also be incorporated into translational studies.

In conclusion, this study establishes a clinically translatable platform that mitigates fibrotic encapsulation and immune rejection of implanted biomaterials and cell-based therapeutics while offering broad potential for addressing foreign body–associated fibrotic diseases. By combining a clinically translatable alginate platform with localized cytokine delivery, we provide a robust and durable solution to implant-associated fibrotic overgrowth and immune rejection in cell therapies. IL-10’s ability to prevent fibrosis and preserve graft functionality underscores its potential as a therapeutic candidate for T1D and other autoimmune diseases. The platform’s capacity to achieve sustained, localized cytokine delivery with minimal systemic toxicity supports both safety and efficacy, marking an advancement over existing immunomodulatory strategies. NHP feasibility and tolerability further support clinical potential. Overall, this work advances a localized immunomodulation strategy to limit foreign body–associated fibrosis, offering opportunities for improving cell therapies, regenerative medicine, and fibrosis-focused therapeutics.

## MATERIALS AND METHODS

### Study design

The objective of this study was to develop and evaluate a localized immunomodulatory platform that uses encapsulated, cytokine-producing cells to prevent immune-mediated rejection and fibrosis in cell-based therapies. We hypothesized that sustained, local delivery of immunoregulatory cytokines, such as IL-10, from alginate-encapsulated engineered RPE cells would attenuate FBR, and preserve the graft. The study included in vitro assessments of cytokine secretion and cell viability, followed by in vivo evaluation in murine and NHP models. For rodent studies, engineered cells were codelivered with human islets and implanted into STZ-induced diabetic C57BL/6J mice to assess fibrosis, glycemic control, and cell function over time. For translational relevance, the platform was further validated in cynomolgus macaques, where encapsulated IL-10–secreting cells were laparoscopically delivered to the peritoneal cavity, and local and systemic immune responses were monitored over time. Capsules were retrieved at various time points for analysis of fibrosis, cell viability, cytokine levels, and immune infiltration using ELISA, live/dead imaging, scRNA-seq, and immunohistochemistry. Additional assays included C-peptide measurements, RT-qPCR, and flow cytometry. Experimental groups were randomly assigned where applicable. Detailed animal numbers, sample sizes, and data points for each experiment are provided in the corresponding figure legends. All animal protocols were approved by the Institutional Animal Care and Use Committees (IACUC) at Rice University (24-069) and the University of Illinois Chicago (23-016) and were conducted in accordance with institutional guidelines.

### Cell culture and transfection

Cell culture and transfection procedures were conducted using materials from Fisher Scientific and Invitrogen, including cell culture media and associated reagents. Expression vectors and helper plasmids were obtained from VectorBuilder. Transfection reagents Lipofectamine 3000 and selection antibiotic (puromycin) were purchased from Invitrogen. The Opti-MEM media for transfection was purchased from Thermo Fisher Scientific. The ARPE-19 cell line, procured from ATCC, underwent regular testing for mycoplasma contamination, yielding negative results. Cells were cultured in Dulbecco’s modified Eagle’s medium (DMEM/F-12) supplemented with 10% fetal bovine serum (FBS) and 1% antibiotic-antimycotic, with media changes occurring three times weekly. ARPE-19 cells were engineered to express cytokines of interest, following the established transfection protocols (*30*). Briefly, ARPE-19 cells were seeded into six-well plates at a cell density of 500,000 cells per well. The cells in the plate were left overnight in the incubator and primed with 2 ml of Opti-MEM serum-free media for 15 min before transfection. A 1:2 ratio of helper plasmid to plasmid expressing cytokine of interest was used to transfect cells. All cell lines were transformed according to the manufacturer’s protocol. After incubation of cells with transfection agents for 4 hours at 37°C, the transfection medium was replaced with fresh culture media, and cells were left to incubate overnight. Then, the cells were selected for expression with puromycin for 2 weeks and expanded to quantify the expression via ELISA.

### Engineered cell encapsulation

For engineered cell encapsulation, SLG20 alginate (UP-LVG, NovaMatrix, Norway) was dissolved at 1.4% (w/v) in 0.8% saline, followed by sterile filtration. Before encapsulation, engineered cells underwent trypsinization and centrifugation at 250*g* for 5 min. Cell pellets were washed twice with Ca-free Krebs buffer and then resuspended in the alginate solution at a density of 5 × 10^6^ cells/ml (∼10,000 cells per capsule). Capsules were produced using a custom-built, two-fluid coaxial electrostatic spraying device (Harvard Apparatus), as described previously (*8*, *30*). Alginate droplets were ejected from an 18-gauge coaxial needle (Rame-Hart) into a barium chloride crosslinking solution, forming hydrogel capsules with a diameter of 1.5 mm. The flow rate of both syringe pumps for core and shell solutions was adjusted to 5 to 6 ml/hour. Capsule size was controlled by adjusting the voltage to between 5.5 and 6 kV (Gamma High Voltage). The capsules were incubated in the crosslinking solution for 15 min, washed with Hepes buffer, and maintained using standard cell culture techniques. For in vivo studies, a low-dose condition consisted of 10,000 cells per 1.5-mm capsule (10 capsules per mouse; 100,000 cells per mouse). A high-dose condition consisted of 40,000 cells per capsule (10 capsules per mouse; 400,000 cells per mouse). In both conditions, 10 large cell-containing capsules were coimplanted with 400 μl of 0.5-mm empty microcapsules per mouse.

### Human islet encapsulation

Human islets (Prodo Labs) were cultured in PIM(S) media from the same source. After centrifugation and washing with Ca-free Krebs buffer, the islets were resuspended in a 1.4% SLG20 solution at a density of 20,000 islet equivalents (IEQ) per 2.7 ml. The islet-containing solution was loaded into a syringe with a 25-gauge blunt-tipped needle. Following the same fabrication procedure as 1.5-mm capsules, microcapsules were adjusted to a size of 0.5 mm by setting the voltage to 10 kV with a flow rate of 200 μl/min. After washing, 400-μl aliquots of microcapsules with 2000 IEQ cells were prepared for implantation. In addition, empty microcapsules, without cells, were fabricated under the same conditions for use in a fibrosis assay.

### Creation of STZ-induced diabetic model

For in vivo studies, a mix of male and female C57BL/6J mice (Charles River Laboratories) aged 8 to 10 weeks was used. All animal studies were approved by Rice University’s IACUC. To induce insulin-dependent diabetes, healthy C57BL/6J mice received STZ treatment. STZ solution at a concentration of 7.5 mg/ml [STZ (50 mg/kg)] was injected into the intraperitoneal space for five consecutive days. BG levels and weights of the mice were measured after a 1-hour fasting. Only mice with BG levels exceeding 350 mg/dl for two consecutive days were deemed diabetic and selected for islet transplantation.

### Intraperitoneal surgical implantation of capsules in mice models

Immunocompetent C57BL/6J mice (8 weeks old) were weighed, anesthetized with 1 to 4% isoflurane in oxygen on a heating pad, and administered subcutaneous Ethiqa XR based on weight. After shaving and sterilizing their abdomens with betadine and isopropanol, a 0.5- to 1-cm midline incision was made through the skin. The peritoneal wall was grasped with forceps, and a 5-mm incision was made along the linea alba. For the fibrosis study (1-, 3-, and 6-month time points), 10 capsules containing engineered cells in a 1.5-mm diameter size were implanted into the intraperitoneal cavity, along with 0.4 ml of empty microcapsules (0.5-mm size). For the scRNA-seq study, 10 capsules containing engineered cells and 0.4 ml of empty microcapsules were implanted into the intraperitoneal space for 7 days. Sham group received an intraperitoneal injection of 1 ml of sterile saline. For the islet study with STZ-induced diabetic mice, portions of empty microcapsules (0.4 ml) were replaced with islet microcapsules (with 2000 IEQ per mouse). The incision was sealed with a suture.

### NHP capsule implantation

Adult Mauritian cynomolgus monkeys were used for capsule implantation studies. All procedures were approved by the University of Illinois Chicago IACUC and adhered to the Guidelines for the Care and Use of Laboratory Animals. Under general anesthesia and sterile conditions, a 2-cm supraumbilical incision was made, and a 5- to 12-mm trocar was inserted to establish pneumoperitoneum with CO_2_ (10 to 14 mmHg). A laparoscopic camera was used for visualization, and a 2-cm incision was made for a second trocar to deliver capsules. A 10 ml of RPE-IL10 capsules were resuspended in 50 ml of saline and administered via a catheter-tip syringe and distributed throughout the intraperitoneal cavity. Incisions were closed in layers using Vicryl sutures (3-0 for muscle, 4-0 for skin). After capsule implantation, the health of the NHPs was closely monitored through regular assessments, including body temperature and weight measurements, as well as comprehensive bloodwork.

### Blood glucose monitoring

Blood glucose levels were monitored three times weekly after the transplantation of islet capsules, conducted without fasting. Mice displaying BG levels below 250 mg/dl were categorized as normoglycemic. Monitoring persisted until all mice reverted to a hyperglycemic state, at which juncture they were euthanized, and the capsules were recovered.

### Static GSIS

To evaluate islet function, static GSIS was performed following coencapsulation. For each condition, either one or three capsules were used per sample. Capsules were first washed twice with KRB buffer [128 mM NaCl, 5 mM KCl, 2.7 mM CaCl_2_, 1.2 mM MgSO_4_, 1 mM Na_2_HPO4, 1.2 mM KH_2_PO_4_, 5 mM NaHCO_3_, 10 mM Hepes, and 0.1% bovine serum albumin (BSA)]. Then, the capsules were incubated in KRB containing 2 mM glucose at 37°C for 1 hour. The solution was then replaced with fresh 2 mM glucose KRB and incubated for an additional hour, after which the supernatant was collected (reported as low glucose on the graph). After that, capsules were incubated in KRB containing 20 mM glucose for 1 hour, and the supernatant was collected (reported as high glucose on the graph). Capsules were washed with fresh KRB between each incubation step. Insulin levels in the collected supernatants were quantified using a human insulin ELISA kit (80-INSHU-E10.1, ALPCO).

### Retrieval of capsules

At a designated point in the implantation period, mice from each study were euthanized using cardiac puncture procedures followed by cervical dislocation. An incision along the abdomen skin and peritoneal wall was made using forceps and scissors. Intraperitoneal fluid and capsules within the cavity were collected using 10 ml of sterile phosphate-buffered saline (PBS). When fibrotic overgrowth was present, capsules were retrieved together with the surrounding newly formed fibrotic tissue. Capsules with minimal overgrowth were typically free-floating and were collected directly. The explanted capsules were washed with Krebs buffer several times, prepared for further imaging, and promptly snap-frozen for future analysis.

### Capsule imaging and cell viability assay

The capsules were gently washed with Krebs buffer and transferred to 35-mm petri dishes for bright- and dark-field imaging using an EVOS microscope. Under ×2 magnification, images were acquired and stitched to observe the entire dish. Fluorescent imaging of cells stained with the live/dead assay (Invitrogen, catalog no. L3224) was performed to assess encapsulated cell viability in both pre- and post-implant capsules. Five capsules from each group underwent washing with PBS and staining with 2 μM calcein AM and 4 μM EthD-1 in complete media. Following a 30-min incubation, capsules were imaged using an EVOS microscope with fluorescence filters. Live cells were visualized with a green fluorescent protein filter in green, while dead cells were observed with a Texas Red filter in red.

### scRNA-seq analysis

On day 7 postimplantation, animals were euthanized, and cell populations from intraperitoneal fluids and retrieved capsules were collected to analyze the local effects of the therapeutic. Spleens from each group were also collected to examine systemic immune cell populations. After removing red blood cells, cells were resuspended in 1 ml of media for cell counting. Propidium iodide was added to stain dead cells following the manufacturer’s instructions. Live cells, sorted at 1000 cells/μl density in DMEM containing 10% FBS, underwent next-generation sequencing at Baylor College of Medicine. Throughout sample processing and cell sorting, cells were maintained on ice. The Single Cell 5’ Gene Expression Library was generated using the Chromium NextGEM Single Cell Immune Profiling Solution 5’v2 protocol by 10x Genomics. The libraries were sequenced on an Illumina NovaSeq 6000 flow cell. Transcripts in each cell were counted using the 10x Cell Ranger 5.0.1 pipeline, with genome mapping to the mm10 genome build using STAR v2.7.2a. The scRNA-seq data were visualized using UMAP embedding in Loupe Browser 5.0 to identify immune cell populations. At this stage, six cell populations were readily apparent. The resulting clusters were assessed for the expression of common immune cell marker genes and were then classified as specific immune cell types based on their expression profiles. The cell identities of each of the six clusters were resolved using the following markers: CD3E (T cells), PRF1 (NK cells), CD19 (B cells), FN1 (monocytes and macrophages), ITGAX (dendritic cells), and LY6G and HDC (granulocytes). These markers assigned individual cell barcodes to their corresponding immune cell type in the Loupe browser. Cell proportions were calculated between cytokines for each immune cell type to assess changes in the infiltrated immune composition. The significance of these changes was calculated using Fisher’s exact test in R (v3.6.1). Differential expression in monocytes between different treatments was derived from the Loupe browser. TGF-β module score was calculated on the basis of expression levels of RPE-IL12–down-regulated genes (Tgfb2, Cdh1, Tgfbr1, Smad7, Mapk3, Tab1, Smad3, Crebbp, and Skil) in BIOCARTA_TGFB_PATHWAY using addModule method in Seurat (v3.1.5). Expression levels of representative genes from the BIOCARTA_TGFB_PATHWAY pathway and HALLMARK_INFLAMMATORY_RESPONSE were *z*-scaled and plotted in a heatmap using ComplexHeatmap (v2.8.0).

### Enzyme-linked immunosorbent assay

For preimplant capsules, a single capsule was added to a 96-well plate (*n* = 8) after encapsulation in 200 μl of culture media at 37°C in a 5% CO_2_-humidified atmosphere. Capsule supernatant was collected after 24 hours. For explant capsules, a single capsule from each mouse post-retrieval was added to a 96-well plate (*n* = 6) in 200 μl of media for 24 hours, and the plate was kept in the incubator. Capsule supernatant was collected from each well and assayed. Capsule supernatants were assayed at 10×, 100×, and 1000× dilutions depending on the ELISA kit. For the local and systemic concentration of cytokines, intraperitoneal fluid was assayed at 1× and 10× dilution, and blood was assayed at 2× and 10× dilution. ELISAs were obtained commercially for mouse-IL10 (R&D Systems, catalog no. M1000B), mIL12 (R&D Systems, catalog no. M1270), mIL2 (R&D Systems, catalog no. M2000). The assay was run according to the manufacturer’s protocols. All the samples were run in duplicates.

### Human C-peptide assay

Following manufacturer protocol, blood from each mouse postexplant was assayed in duplicate without dilution (ALPCO, catalog no. 80-CPTHU-E01.1). All the standards were also assessed in duplicates.

### Immunofluorescence staining for confocal imaging

Retrieved microcapsules were washed with Krebs buffer, fixed in 4% paraformaldehyde overnight at 4°C, and permeabilized with 1% Triton X-100. Following blocking with a 1% BSA solution, samples were incubated with antibody cocktails [diluted at 1:200 Alexa Fluor 488 anti-mouse CD68 antibody (catalog no. 137012, BioLegend), 1:200 anti-mouse α–smooth muscle–Cy3 (catalog no. C6198, Sigma-Aldrich), and 4′,6-diamidino-2-phenylindole (catalog no. R37606, Invitrogen) in 1% BSA] for 1 hour at room temperature. After washing, samples were transferred for imaging using a Nikon A1-Rsi confocal microscope.

### RT-qPCR analysis

Total RNA was extracted from 100 μl of retrieved microcapsules (empty capsules with a 0.5-mm size) using the RNeasy Mini Kit (catalog no. 74104, QIAGEN). The extracted RNA was converted to cDNA for RT-qPCR using the high-capacity cDNA reverse transcription kit (catalog no. 4368814, Applied Biosystems). Real-time qPCR (Bio-Rad) was performed using SYBR Green (catalog no. A25742, Applied Biosystems), and reactions were run in triplicates under specified conditions. Data analysis was conducted using the 2^−ΔΔCT^ method, comparing relative RNA levels after normalization to mouse ActB and empty cap control. The primer details are listed in table S7.

### Flow cytometry

All antibodies were commercially sourced, prepared fresh on the day of staining, and stored in the dark at 4°C or on ice to preserve their efficacy. Cells were prepared for staining for flow cytometry as previously reported (*30*). Samples were washed with 200 μl of cell staining buffer (catalog no. 420201, BioLegend) before staining. For NHP samples, cells were stained with anti-human CD4 (1:200; catalog no. 555349, BD) and anti-human CD25 (1:100; catalog no. 560920, BD) for 20 min on ice in the dark. After staining, samples were washed three times with cell staining buffer and fixed for an hour at room temperature with fixation buffer (catalog no. 420801, BioLegend). After fixation, cells in each sample were resuspended in cell staining buffer and run through a 40-μm filter before acquisition on a Sony MA900. Single-color, fluorescence minus one (FMO) controls, as well as a negative unstained control, were prepared for each analysis. Gating method was described in the fig. S11. For mouse samples, cells were stained with viability dye (1:1000; catalog no. 423114, BioLegend), anti-mouse CD11b (1:50; 101224, BioLegend), anti-mouse CD206 (1:40; catalog no. 141716, BioLegend), and anti-mouse CD86 (1:20; catalog no. 105014, BioLegend) for 30 min on ice in the dark. Then, cells were washed with fluorescence-activated cell sorting (FACS) buffer and fixed with fixation buffer (catalog no. 88-8824-00, Thermo Fisher Scientific) for 30 min at room temperature. After fixation, cells in each sample were resuspended in 300 μl of FACS buffer and run through a 40-μm filter for eventual analysis using an SA3800 Spectral Analyzer. Single-color, unstrained control and FMOs were included. The gating strategy is shown in the fig. S12.

### Histology

The Baylor Pathology and Histology Core facilitated the processing, sectioning, and histological analysis of NHP organs. Samples were paraffin-embedded, excess paraffin was meticulously trimmed, and sagittal sections were prepared and stained with H&E for detailed examination. Masson’s trichrome staining on microcapsules was performed by MD Anderson Research Histology Core.

### Statistical analysis

All statistical analyses were conducted with GraphPad Prism 9. One-way or two-way analysis of variance (ANOVA) with Bonferroni multiple-comparison correction was used to determine *P* values (****P < 0.0001, ***P < 0.0002, **P < 0.002, and *P < 0.033).
