# Supplementary material for: Localized immunomodulation with cytokine-producing cells to mitigate foreign body responses in rodents and a nonhuman primate
Source: Sci Adv. 2026 Aug 5;12(32):eaec7053. doi: 10.1126/sciadv.aec7053 (PMC13440420; doi:10.1126/sciadv.aec7053)
Supplement: Supplementary file 1 [file sciadv.aec7053_sm.pdf]

Science Advances
AAAS

Supplementary Materials for

Localized immunomodulation with cytokine-producing cells to mitigate foreign body responses in rodents and a nonhuman primate

Boram Kim *et al.*

Corresponding author: Omid Veiseh, omid.veiseh@rice.edu

Sci. Adv. **12**, eaec7053 (2026)
DOI: 10.1126/sciadv.aec7053

The PDF file includes:

Figs. S1 to S12
Tables S1, S6 and S7
Legends for tables S2 to S5

Other Supplementary Material for this manuscript includes the following:

Tables S2 to S5

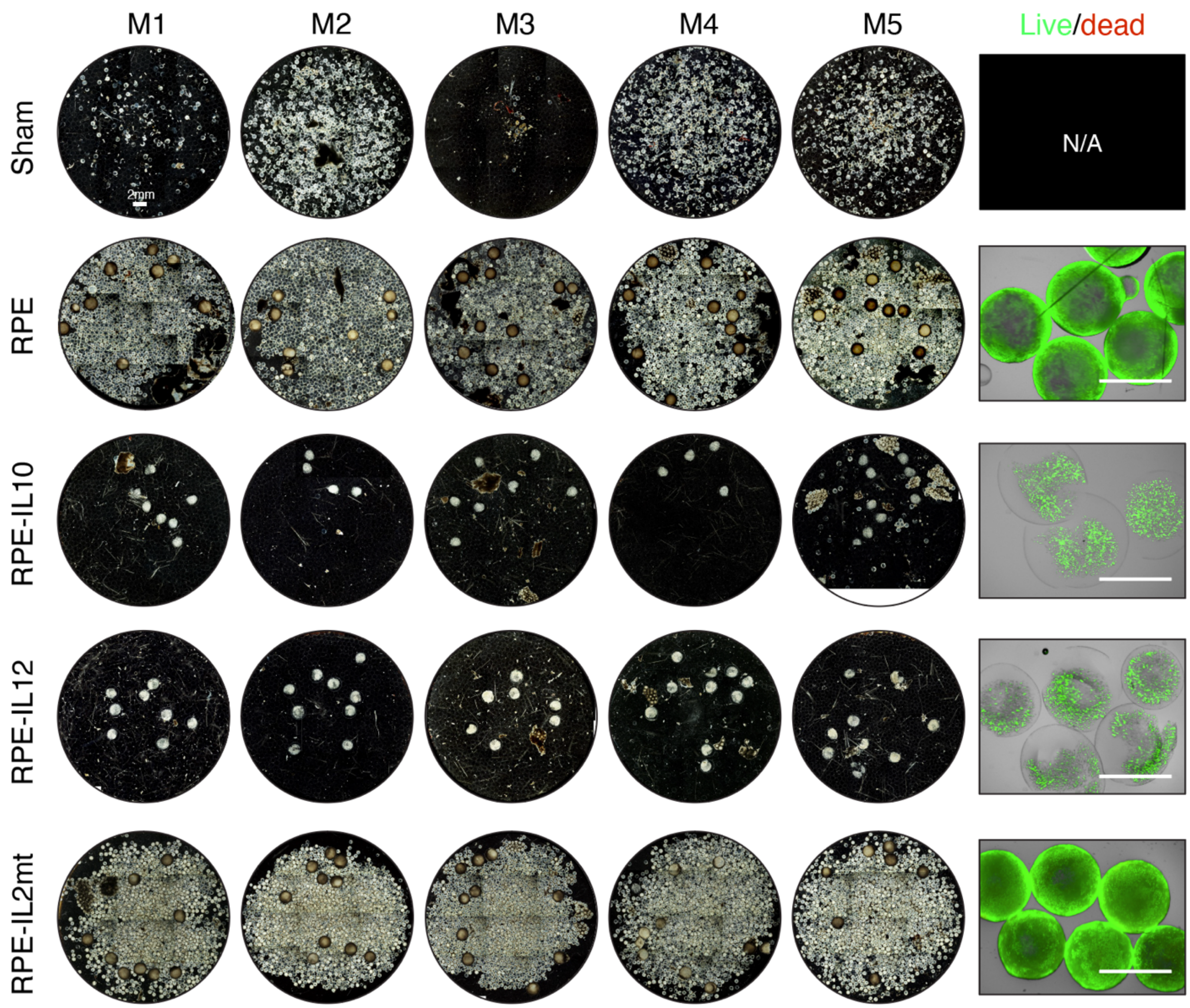

Fig. S1. Short-term effect of cytokine-producing capsules in preventing fibrosis. Dark-field images of explanted capsules (low-dose; 10K cells per capsule) from individual mouse post one month IP implants. The last column shows the representative live/dead staining images of explanted RPE capsules (Live, green; Dead, red). Scale bar, 2mm.

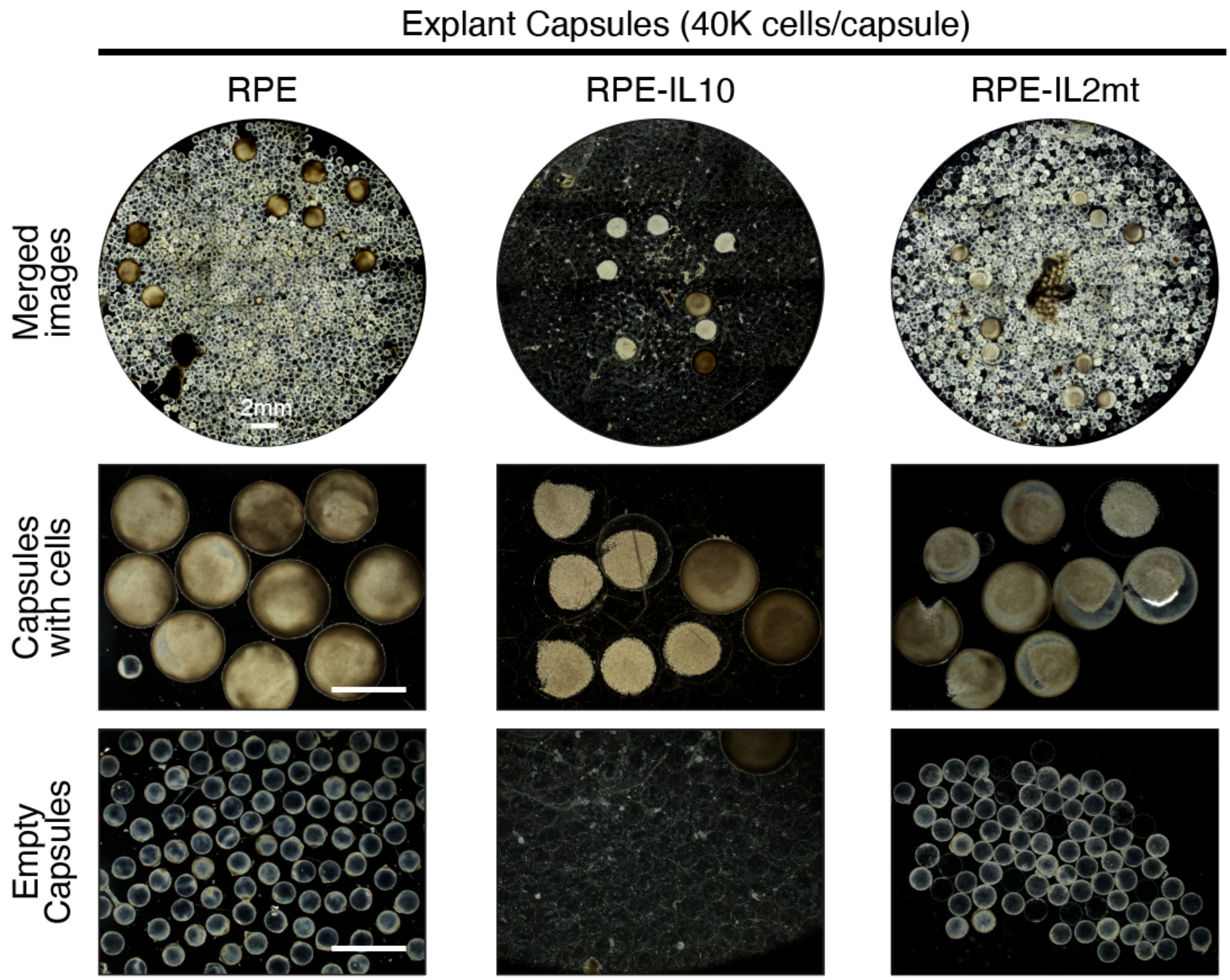

Fig. S2. Short-term effect of high-dose cytokine-producing capsules on fibrosis. Capsules were fabricated at 40,000 cells per capsule (high-dose condition). Representative dark-field images of explanted capsules post one month IP implants. Scale bar, 2mm.

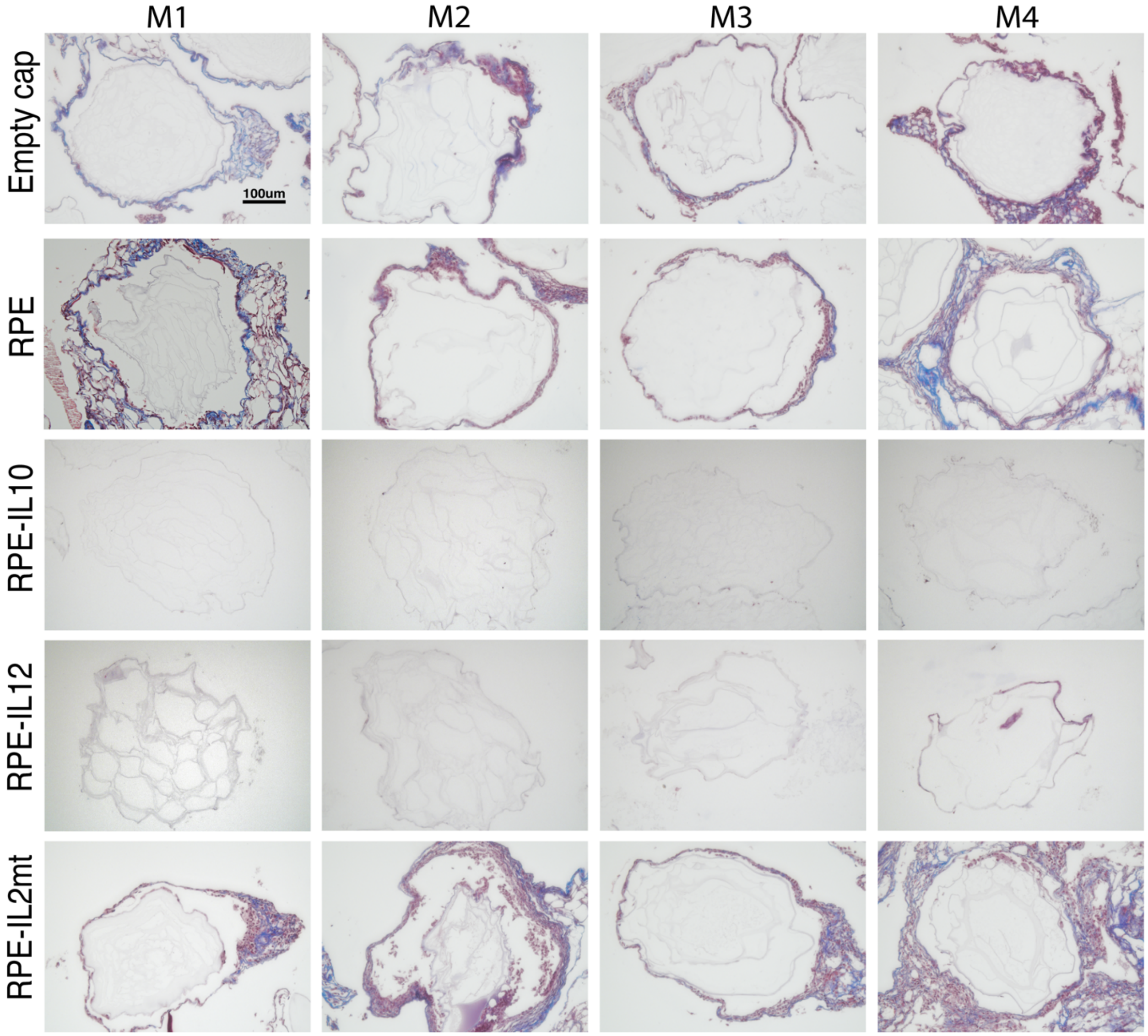

Fig. S3. Masson's Trichrome on microcapsules retrieved from mice after 1 month. Representative images of microcapsules from each mouse imaged at 20x magnification.

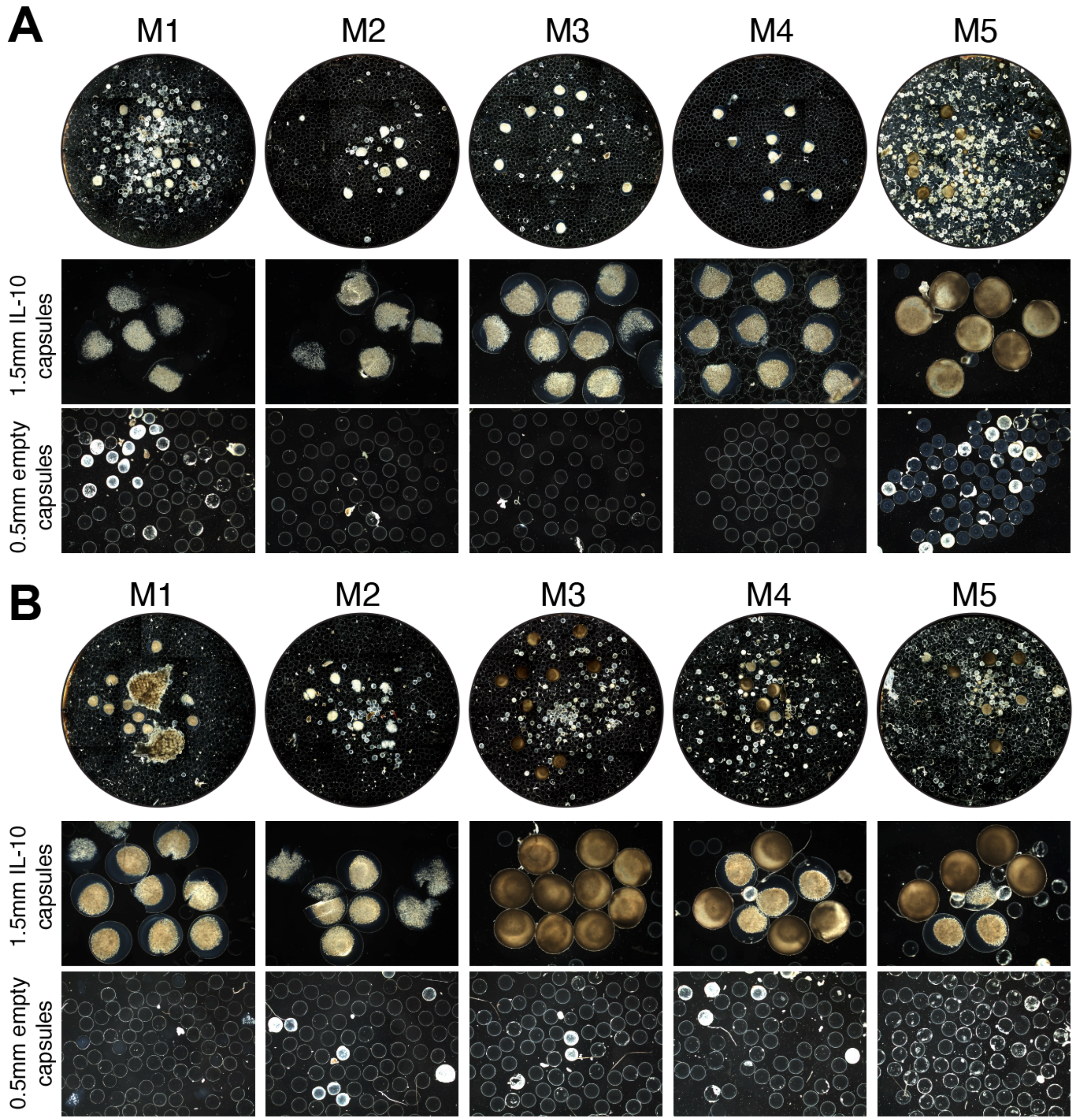

Fig. S4. Long-term effect of RPE-IL10 in preventing fibrosis. (**A** and **B**) Dark-field images of retrieved capsules after three months (A) and six months (B) implantation.

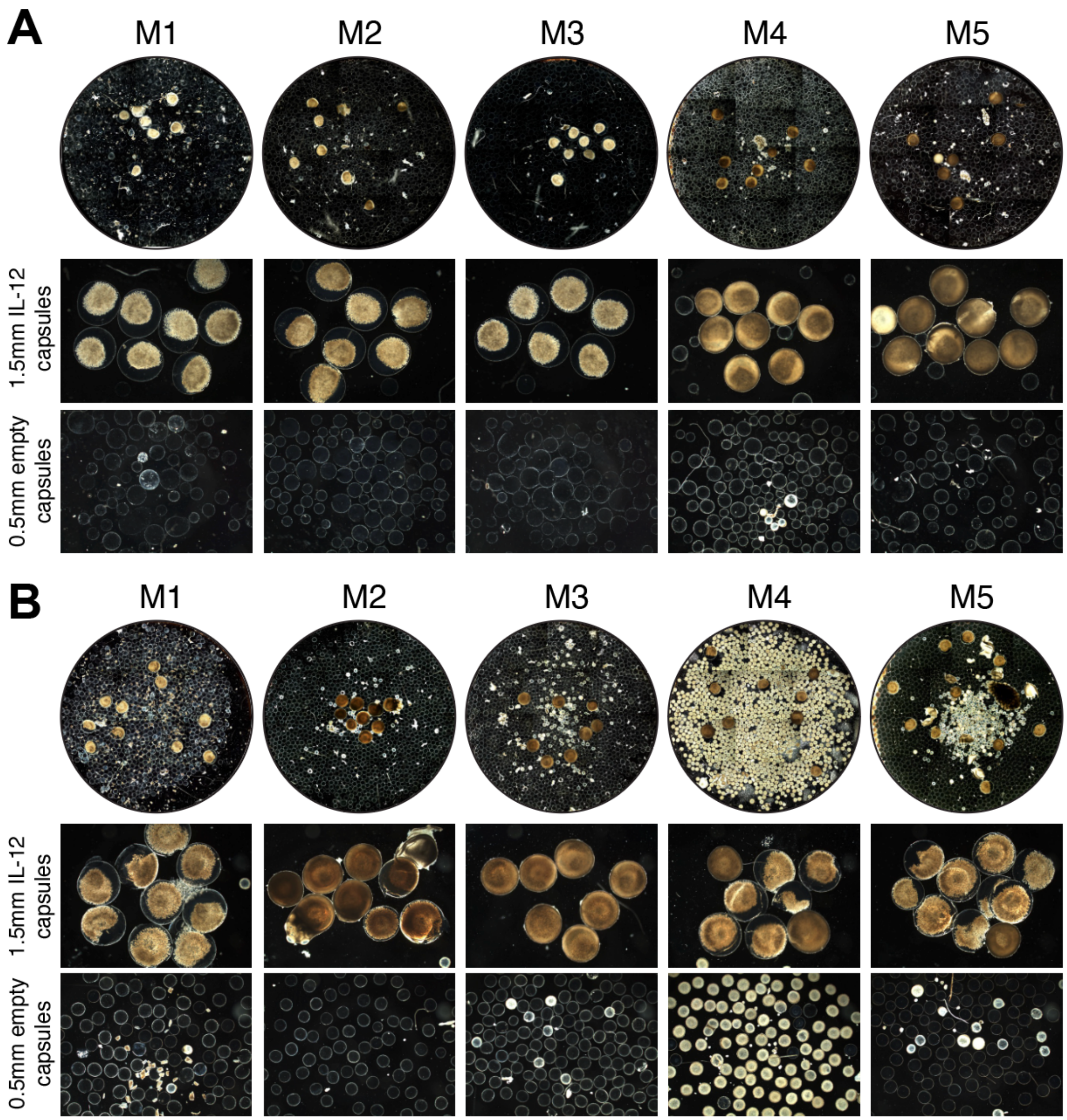

Fig. S5. Long-term effect of RPE-IL12 in preventing fibrosis. (**A** and **B**) Dark-field images of retrieved capsules after three months (A) and six months (B) implantation.

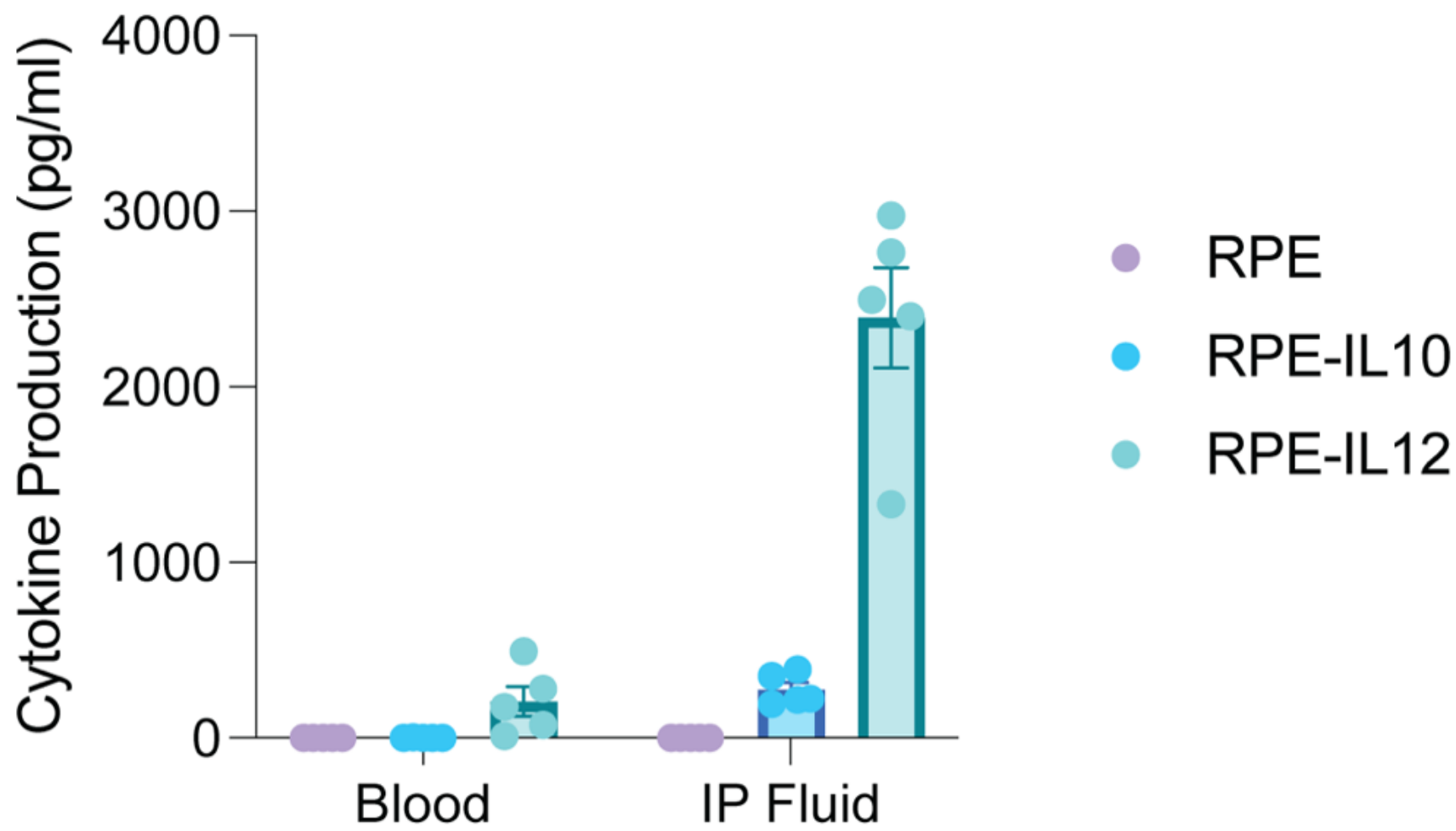

Fig. S6. Short-term cytokine secretion levels. Cytokine concentrations in the intraperitoneal (IP) fluid and blood at day 7 post implantation.

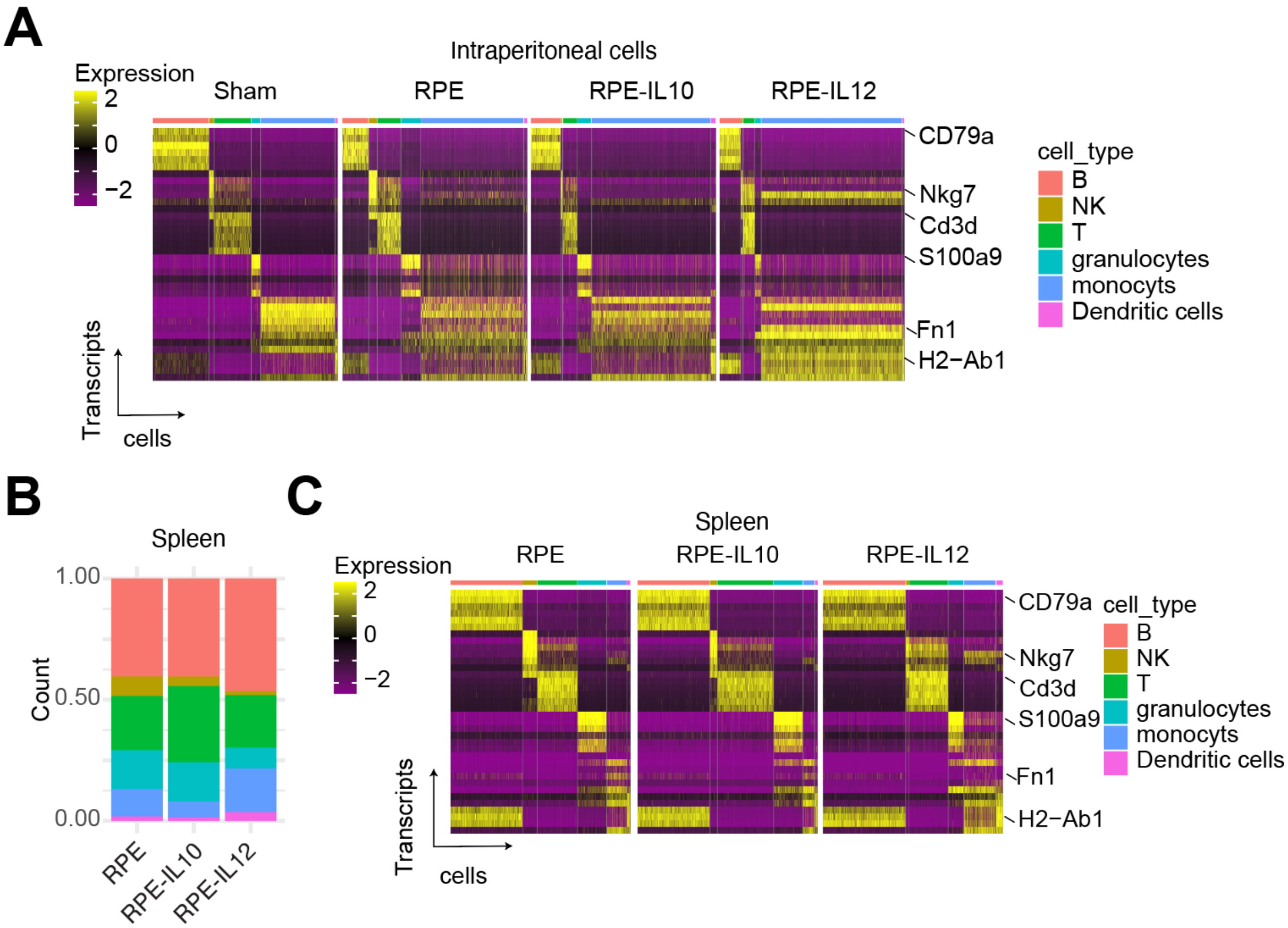

Fig. S7. scRNA-seq analysis. (**A**) Single-cell transcriptional profiles of markers used to define each cell immune cell type in intraperitoneal cells. (**B**) Composition of splenic immune cell identities. (**C**) Single-cell transcriptional profiles of markers used to define each cell immune cell type in splenic cells.

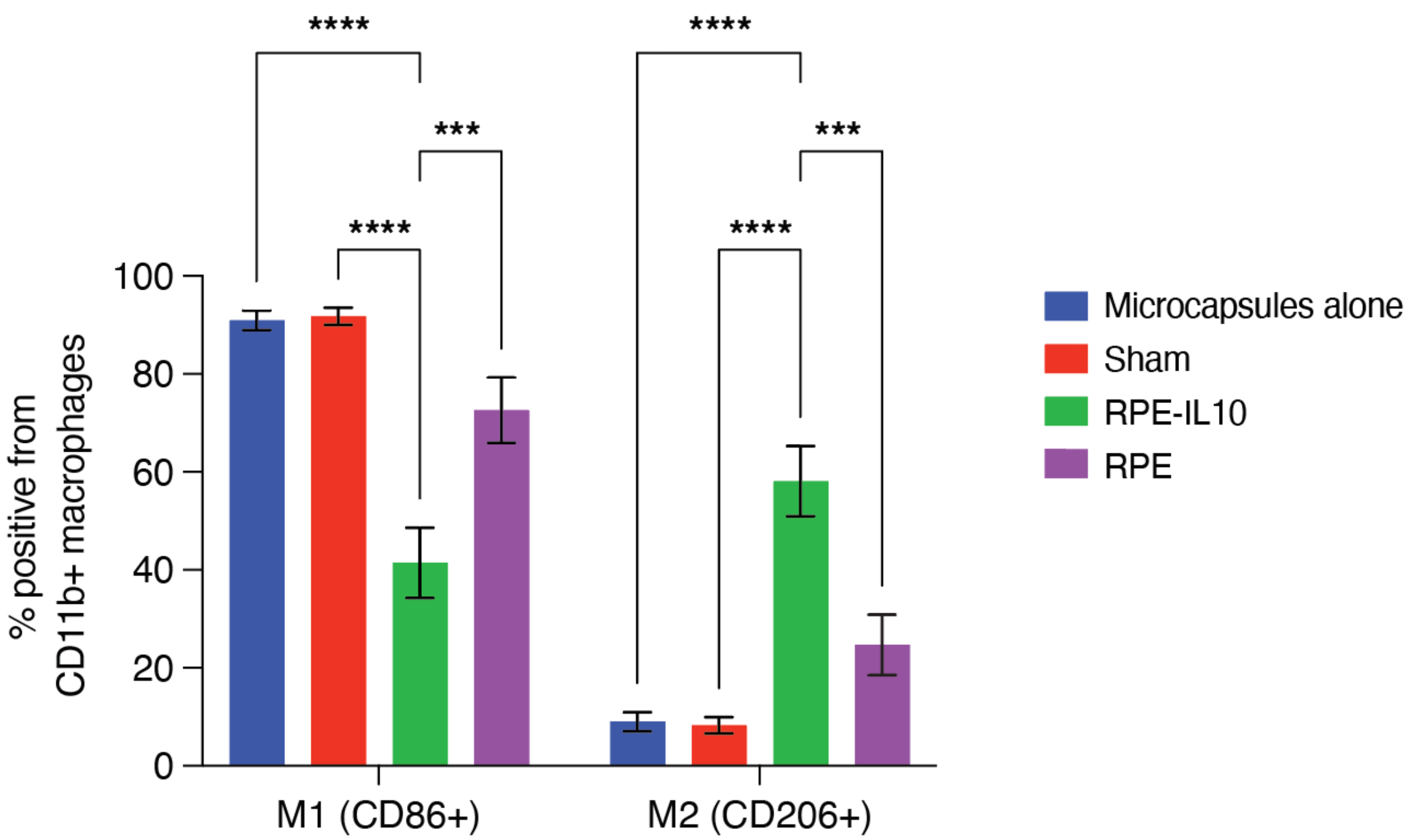

Fig. S8. Flow cytometry on intraperitoneal cells of healthy mice at Day 7 post-implantation. The frequency of M1-like macrophages (CD11b+CD86+) versus M2-like macrophages (CD11b+CD206+) was evaluated in the intraperitoneal space among the groups.

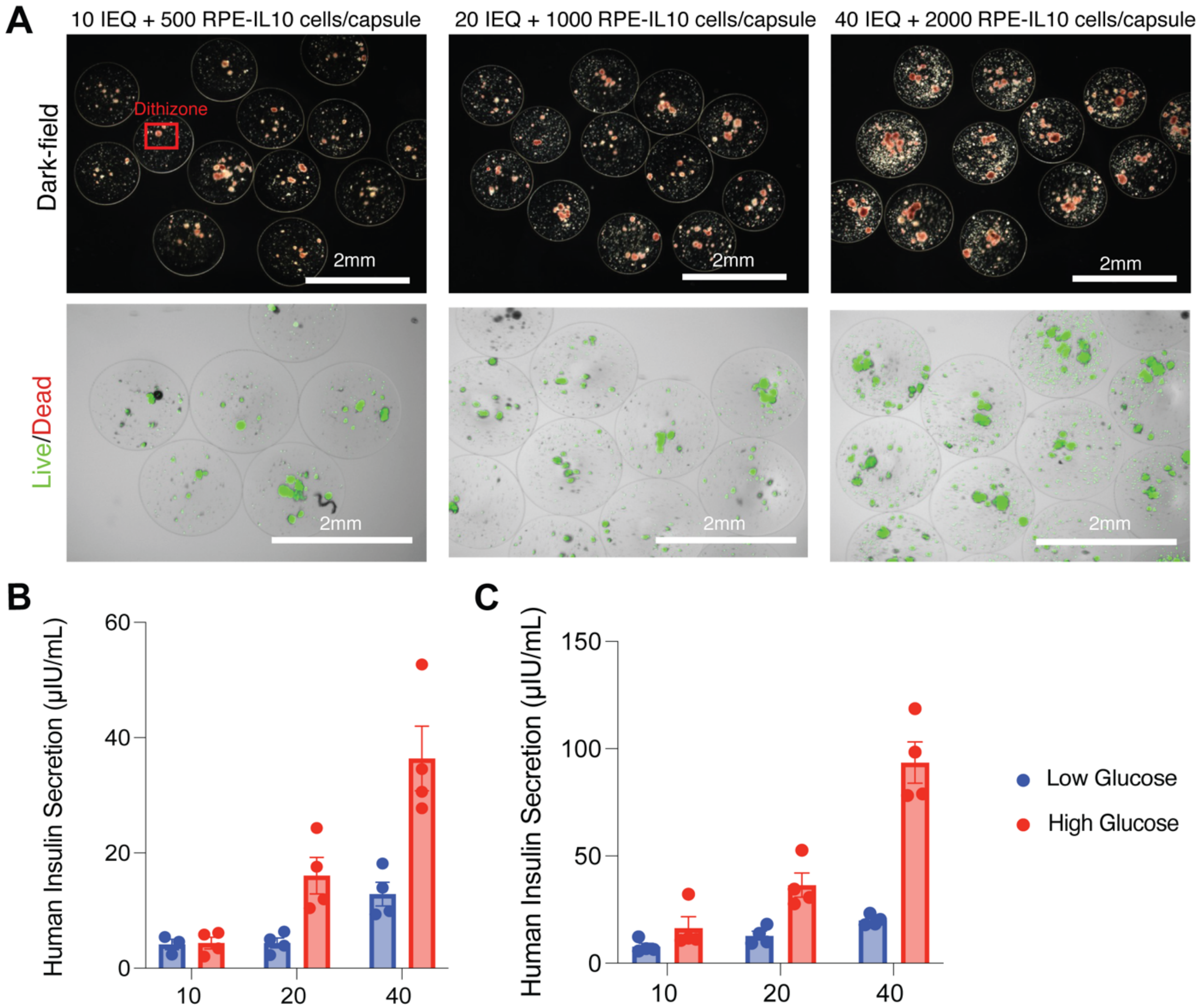

Fig. S9. Validation of co-encapsulation of human islets and RPE-IL10 cells in vitro. (**A**) Viability staining confirms cell survival (Live; green, Dead; red), while dithizone staining specifically highlights the structural integrity of encapsulated islets. (**B** and **C**) Static GSIS was performed in vitro following encapsulation to assess human insulin secretion across different islet-to-RPE-IL10 cell ratios, 10 (10 IEQ and 500 RPE-IL10 cells/capsule), 20 (20 IEQ and 1000 RPE-IL10 cells/capsule) and 40 (40 IEQ and 200 RPE-IL10 cells/capsule). (B) represents results from single-capsule samples, while (C) shows data from three-capsule samples per condition.

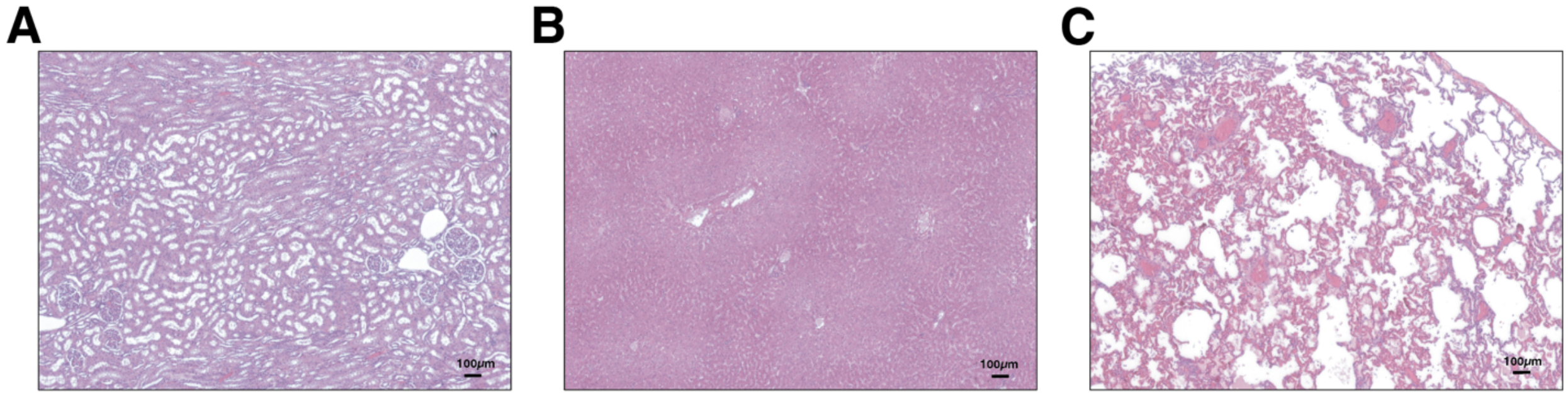

Fig. S10. NHP histology images. (**A** to **C**) Representative histology images of NHP kidney (A), liver (B), and lung (C) taken at 20x magnification. No notable clinical abnormalities were detected.

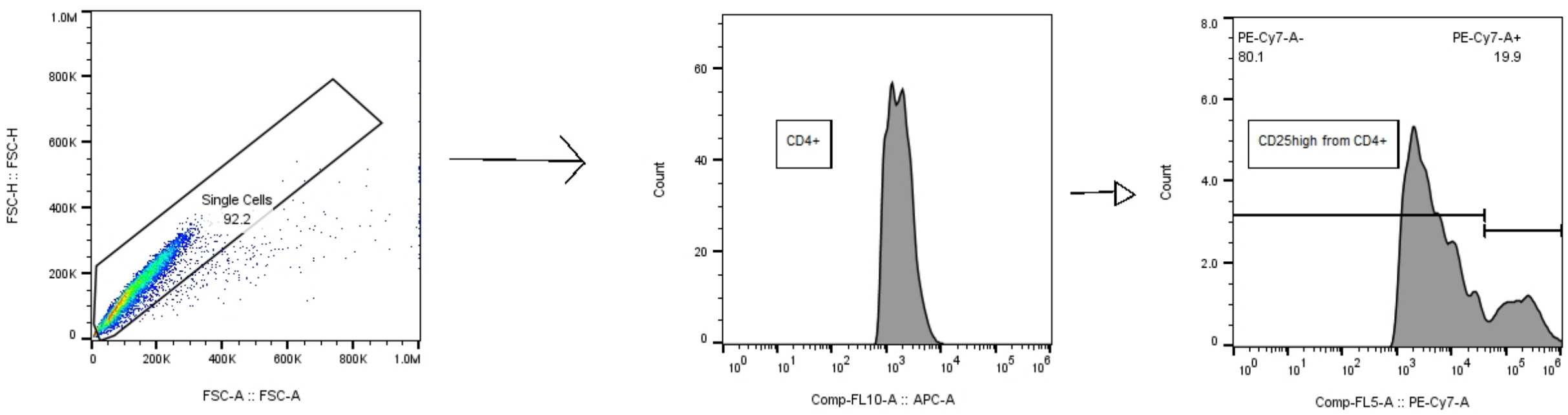

Fig. S11. NHP flow cytometry gating strategy.

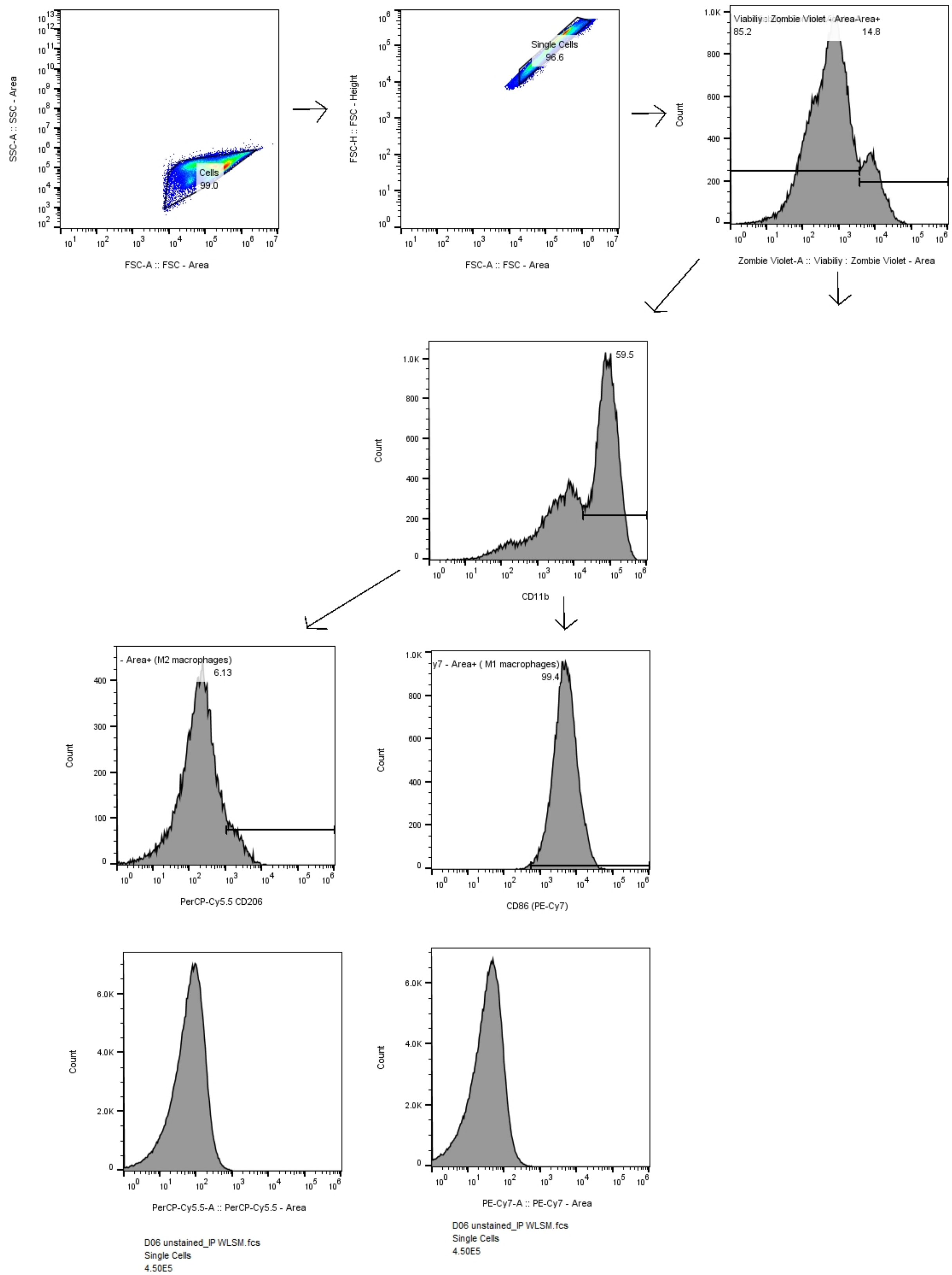

Fig. S12. Flow cytometry gating strategy on mice samples.

Table S1. Composition of immune infiltrate in local vs. systemic space.

Local: Percentage of immune cells in IP/capsule (%)												
**Cell type**	**B**	**Other**	**DC**	**Other**	**NK**	**Other**	**T**	**Other**	**Granulo**	**Others**	**Mono**	**Other**
RPE	14.3	85.7	1.8	98.2	4.5	95.5	12.8	87.2	10.6	89.4	56.1	43.9
IL-10	16.2	83.8	2.5	97.5	0.8	99.2	7.8	92.2	7.6	92.4	65.1	34.9
RPE	14.3	85.7	1.8	98.2	4.5	95.5	12.8	87.2	10.6	89.4	56.1	43.9
IL-12	11.6	88.4	1.1	98.9	1	99	6.2	93.8	3.2	96.8	77.0	23.0
**Systemic: Percentage of immune cells in spleen (%)**												
**Cell type**	**B**	**Other**	**DC**	**Other**	**NK**	**Other**	**T**	**Other**	**Granulo**	**Others**	**Mono**	**Other**
RPE	40.5	59.5	1.9	98.1	8.1	91.9	22.4	77.6	16.1	83.9	11.1	88.9
IL-10	40.5	59.5	1.5	98.5	3.9	96.1	31.5	68.5	16.2	83.8	6.4	93.6
RPE	40.5	59.5	1.9	98.1	8.1	91.9	22.4	77.6	16.1	83.9	11.1	88.9
IL-12	46.5	53.5	3.7	96.3	1.7	98.3	21.7	78.3	8.6	91.4	17.8	82.2

* B, B cells; DC dendritic cells; NK, NK cells; T, T cells; Granulo, Granulocyte; Mono, monocytes.

Table S2. Gene set enrichment analysis of differential analysis between RPE-IL12 vs. RPE in intraperitoneal monocytes.

Table S3. Gene set enrichment analysis of differential analysis between RPE-IL10 vs. RPE in intraperitoneal monocytes.

Table S4. Gene set enrichment analysis of differential analysis between RPE-IL10 vs. RPE in splenic monocytes.

Table S5. Gene set enrichment analysis of differential analysis between RPE-IL12 vs. RPE in splenic monocytes.

Note: Tables S2-S5 are provided as one merged .xlsx file.

Table S6. NHP hematological data for the primates given RPE-IL10 capsules.

Units / Days	RBCs (x10^6/μL)	WBCs (x10^3/μL)	Lymphocytes count (x10^3/μL)	Platelet count (x10^3/μL)
Day 0	6.69	8.35	73.7	427
Day 1	6.33	10.06	35.3	349
Day 4	5.15	13.57	44.4	191
Day 7	4.15	12.52	48.8	546
Day 14	3.29	12.83	61.3	529
Day 21	4.85	12.49	49.1	586
Day 28	5.89	7.8	58.4	628

Table S7. List of RT-qPCR primers.

Target	Primers (5'→3')
Mouse Alpha Smooth muscle actin (αSMA)	Forward: CGCTTCCGCTGCCCAGAGACT
	Reverse: TATAGGTGGTTTCGTGGATGCCCGCT
Mouse Collagen 1a1 (Col1a1)	Forward: CATGTTCAGCTTTGTGGACCT
	Reverse: GCAGCTGACTTCAGGGATGT
Mouse Collagen 1a2 (Col1a2)	Forward: GCAGGTTCACCTACTCTGTCCT
	Reverse: CTTGCCCCATTCATTTGTCT
Mouse β-actin (ActB)	Forward: GCTTCTTTGCAGCTCCTTCGTT
	Reverse: CGGAGCCGTTGTCGACGACC
